# TRPC3 Regulates Islet Beta‐Cell Insulin Secretion

**DOI:** 10.1002/advs.202204846

**Published:** 2023-01-15

**Authors:** Gaëlle Rached, Youakim Saliba, Dina Maddah, Joelle Hajal, Viviane Smayra, Jules‐Joel Bakhos, Klaus Groschner, Lutz Birnbaumer, Nassim Fares

**Affiliations:** ^1^ Physiology and Pathophysiology Research Laboratory Pole of Technology and Health Faculty of Medicine Saint Joseph University of Beirut POBox. 17‐5208 ‐ Mar Mikhaël Beirut 1104 2020 Lebanon; ^2^ Faculty of Medicine Saint Joseph University Saint Joseph University of Beirut POBox. 17‐5208 ‐ Mar Mikhaël Beirut 1104 2020 Lebanon; ^3^ Gottfried‐Schatz‐Research‐Centre‐Biophysics Medical University of Graz Graz 8010 Austria; ^4^ School of Medical Sciences Institute of Biomedical Research (BIOMED) Catholic University of Argentina Buenos Aires C1107AAZ Argentina; ^5^ Signal Transduction Laboratory National Institute of Environmental Health Sciences Research Triangle Park Durham NC C1107AAZ USA

**Keywords:** beta cells, calcium, diabetes, insulin, TRPC3

## Abstract

Insulin release is tightly controlled by glucose‐stimulated calcium (GSCa) through hitherto equivocal pathways. This study investigates TRPC3, a non‐selective cation channel, as a critical regulator of insulin secretion and glucose control. TRPC3's involvement in glucose‐stimulated insulin secretion (GSIS) is studied in human and animal islets. TRPC3‐dependent in vivo insulin secretion is investigated using pharmacological tools and *Trpc3^−/−^* mice. TRPC3's involvement in islet glucose uptake and GSCa is explored using fluorescent glucose analogue 2‐[*N*‐(7‐nitrobenz‐2‐oxa‐1,3‐diazol‐4‐yl) amino]‐2‐deoxy‐D‐glucose and calcium imaging. TRPC3 modulation by a small‐molecule activator, GSK1702934A, is evaluated in type 2 diabetic mice. TRPC3 is functionally expressed in human and mouse islet beta cells. TRPC3‐controlled insulin secretion is K_ATP_‐independent and primarily mediated by diacylglycerol channel regulation of the cytosolic calcium oscillations following glucose stimulation. Conversely, glucose uptake in islets is independent of TRPC3. TRPC3 pharmacologic inhibition and knockout in mice lead to defective insulin secretion and glucose intolerance. Subsequently, TRPC3 activation through targeted small‐molecule enhances insulin secretion and alleviates diabetes hallmarks in animals. This study imputes a function for TRPC3 at the onset of GSIS. These insights strengthen one's knowledge of insulin secretion physiology and set forth the TRPC3 channel as an appealing candidate for drug development in the treatment of diabetes.

## Graphical abstract

TRPC3, a non‐selective calcium channel, is identified as a regulator of glucose‐stimulated insulin release in human and mouse beta cells via a K_ATP_‐independent mechanism. Small‐molecule TRPC3 modulation improves insulin secretion and diabetic hallmarks in animals. These findings advance the understanding of insulin secretion physiology and render the TRPC3 channel an appealing diabetes treatment target.

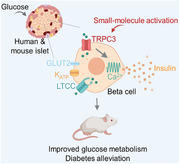

## 1 Introduction

Insulin secretion is primarily triggered by extracellular glucose levels and occurs in two phases:^[^ ^1^ ^]^ the first is rapid and lasts less than 10 min and the second allows for a gradual oscillatory and sustained rise in insulin secretion in response to the persistent elevated glucose blood concentrations and lasts until euglycemia is achieved. The first phase mechanisms involve potassium and calcium channels leading to abrupt intracellular calcium rise, whereas the second phase mechanisms are still largely under‐explored but likely involve multiple ion channels and complex signaling pathways, leading to intracellular calcium oscillations.^[^ ^1^, ^2^, ^3^, ^4^ ^]^ Thus, intracellular calcium is the sole driver of the initial acute insulin secretion phase^[^ ^5^ ^]^ and seems to be indispensable in the later oscillatory stages.

The transient receptor potential (TRP) superfamily of ion channels is pivotally involved in a wide range of both physiological and pathological conditions.^[^ ^6^ ^]^ TRP channels, especially transient receptor potential melastatin TRPM2, 3, 4, and 5, and canonical TRPC1, reportedly play a role in regulating insulin secretion.^[^ ^7^ ^]^ A decline in intracellular calcium concentration and insulin secretion in response to glucagon‐like peptide 1 (GLP‐1) has been observed in TRPM2 knockout mice.^[^ ^8^ ^]^ TRPM4 and TRPM5 contribute to the regulation of insulin secretion by triggering Na^+^ entry in response to an initial increase in intracellular calcium through voltage‐gated calcium channels (VDCCs).^[^ ^7^ ^]^ The TRPM5 channel mediates insulin secretion in response to extracellular glucose, GLP‐1, and a high concentration of L‐arginine.^[^ ^9^ ^]^ Activation of TRPM3 by external steroid signals also enhances intracellular calcium flow.^[^ ^7^ ^]^ Furthermore, TRPC1 is involved in the action of acetylcholine on glucose‐stimulated insulin secretion (GSIS) via store‐operated calcium entry regulation in beta cells.^[^ ^10^ ^]^

The transient receptor potential canonical 3 (TRPC3) channels are non‐selective cation channels that belong to the canonical subfamily.^[^ ^7^ ^]^ TRPC3 channels participate in physiological functions^[^ ^11^ ^]^ and are also involved in a variety of diseases.^[^ ^12^, ^13^, ^14^ ^]^ TRPC3 channels have proven function in the pancreas but only in the exocrine acini.^[^ ^15^ ^]^ As for the endocrine pancreas, while these channels have been functionally implicated in beta‐cell proliferation^[^ ^16^ ^]^ and GPR40‐mediated insulin secretion,^[^ ^17^ ^]^ they are present at very low expression levels,^[^ ^18^ ^]^ and others have even casted a doubt on TRPC3 existence in such cells based on RNA‐seq studies.^[^ ^19^, ^20^, ^21^ ^]^ Thus, their existence and functional relevance in beta cells is uncertain and has yet to be established.

Using healthy and diabetic mouse models as well as human pancreatic tissue, we sought to determine whether TRPC3 channels are involved in insulin secretion and analyze the underlying cellular mechanisms. Here we unravel TRPC3 as a key component of beta cell function and propose this channel as a potential target for antidiabetic interventions.

## 2 Results

### 2.1 TRPC3 Channels Are Present at a Low Level in Human and Mouse Pancreatic Langerhans Islets

**Figure 1:**
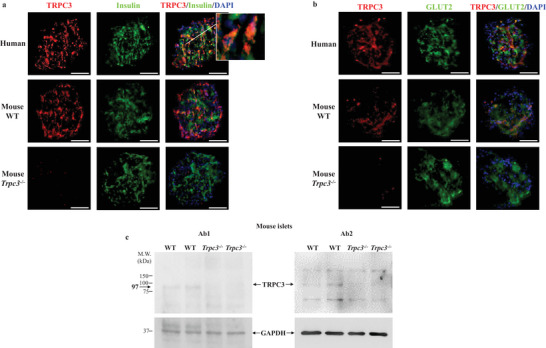
TRPC3 channels are expressed at a low level in human and mouse pancreatic beta cells. a) Insulin and TRPC3 fluorescence images from human and mouse (WT and *Trpc3^−/−^*) islets. The inset is a higher magnification of the regional selection; the orange color in the individual beta cells represents the colocalization of insulin and TRPC3 around the nuclear DAPI staining. b) GLUT2 and TRPC3 fluorescence images from human and mouse (WT and *Trpc3^−/−^*) islets. Scale bars: 50 µm. c) Validation of the modest TRPC3 presence in isolated mouse islets using a monoclonal (Ab1) and a polyclonal (Ab2) antibodies. The specific TRPC3 band appears at the predicted molecular weight of 97 kDa. GAPDH was used as an internal control. *n* = 3 western blots for each condition.

TRPC channels have been notoriously difficult to detect in western blots, with several off‐targets detected by many antibodies used in literature.^[^ ^22^ ^]^ We used a previously generated and validated monoclonal anti‐TRPC3 antibody^[^ ^23^ ^]^ and verified it using *Trpc3^−/−^* tissues and isolated islets. Human and mouse islets stained positive for TRPC3 and insulin as well as glucose transporter 2 (GLUT2). TRPC3 was weakly expressed in beta cell regions, and double immunofluorescence revealed colocalization with insulin and GLUT2, a beta cell‐specific transporter (**Figure** **1**a,b). Additionally, the immunofluorescence indicated the absence of TRPC3 in *Trpc3^−/−^* islets, supporting the specificity of the monoclonal TRPC3 antibody (Figure 1a,b). TRPC3 antibody (Ab1) validation was further carried out by western blot analysis in isolated islets, and revealed TRPC3 channel protein expression at a low level in wild‐type (WT) but not in *Trpc3^−/−^* mice. The monoclonal anti‐TRPC3 antibody detected the protein at the predicted 97 kDa molecular weight, and the specific TRPC3 band was hardly noticeable in *Trpc3^−/−^* tissues (Figure 1c). The presence of TRPC3 at a low level in WT islets was also confirmed using a second polyclonal TRPC3 antibody (Ab2) (Figure 1c).

### 2.2 TRPC3 Channel Inhibition and Knock Out Lead to Glucose Intolerance in Mice by Decreasing Insulin Secretion

**Figure 2:**
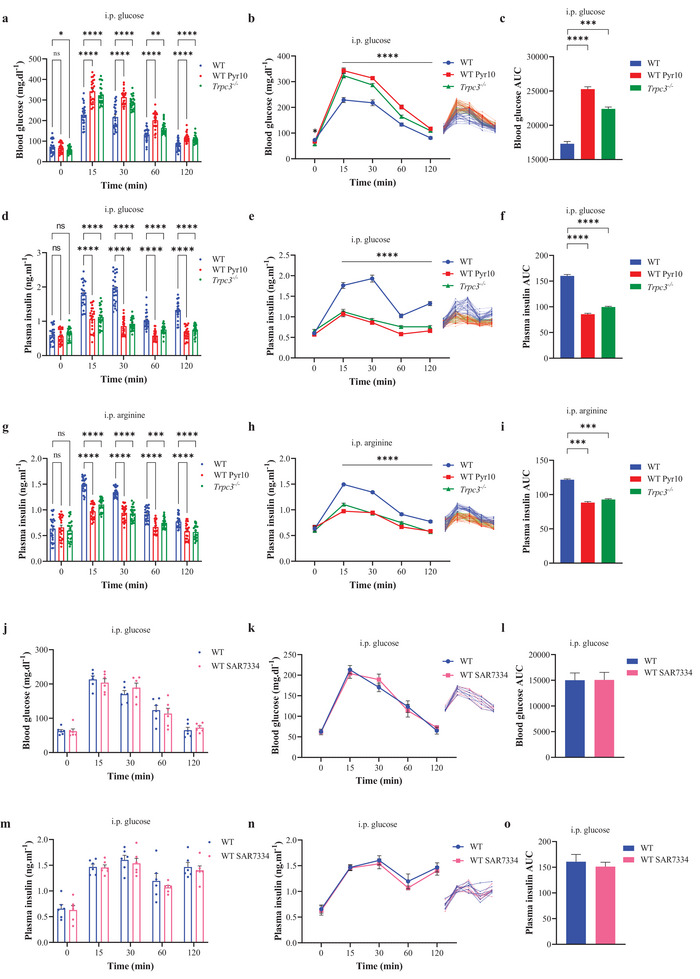
Pharmacologic inhibition and knock out of TRPC3 induce glucose intolerance in mice. a) Raw scatter plots of plasma glucose concentrations during IpGTT. b) Curve representation of the average plasma glucose concentrations during IpGTT. On the right: curve representation of raw plasma glucose concentration data. c) Area under curve (AUC) of the average plasma glucose concentrations in (b). TRPC3 was inhibited by Pyr10. d) Raw scatter plots of plasma insulin concentrations during IpGTT. e) Curve representation of the average plasma insulin concentrations. On the right: curve representation of raw plasma insulin concentration data. f) AUC of the average plasma insulin concentrations in (e). g) Raw scatter plots of plasma insulin concentrations during arginine tolerance test. h) Curve representation of the average plasma insulin concentrations during i.p. arginine tolerance test. On the right: curve representation of raw plasma insulin concentration data. i) AUC of the average plasma insulin concentrations in panel h. *n* = 25 mice in each condition. *p < 0.05, **p < 0.01, ***p < 0.001, and ****p < 0.0001 versus WT; ns: non‐significant. j–o) Raw scatter plots of plasma glucose and insulin concentrations, curve representation of the average and raw plasma glucose and insulin, and area under curve of the average plasma glucose and insulin concentrations during IpGTT w/o TRPC6 inhibition by SAR7334. *n* = 6 mice in each condition. Repeated measures two‐way ANOVA were used followed by either Dunnett's tests in (a), (b), (d), (e), (g), (h), (m), and (n) or Sidak's tests in (j) and (k). One‐way ANOVA were used followed by Tukey's tests in (c), (f), (i), (l), and (o).

Intraperitoneal glucose tolerance tests (IpGTT), a cornerstone test in insulin research, was used to compare the glucose tolerance of mice given the selective TRPC3 inhibitor N‐[4‐[3,5‐Bis(trifluoromethyl)‐1H‐pyrazol‐1‐yl]phenyl]‐4‐methyl‐benzenesulfonamide (Pyr10) and of *Trpc3^−/−^* animals to normal WT mice. Pyr10 is a pyrazole drug with high selectivity for TRPC3 but no effect on other TRPC isoforms.^[^ ^24^, ^25^ ^]^ One study reported an effect of Pyr10 on TRPC6,^[^ ^26^ ^]^ however, an overexpression system was used which might affect the function and behavior of TRPC channels, as previously described in such systems for TRPC3.^[^ ^27^ ^]^ During IpGTT, WT mice treated with Pyr10 (WT Pyr10) and *Trpc3^−/−^* mice showed higher blood glucose concentrations at T15 compared to WT mice (**Figure** **2**a). From T15 till T120, higher blood glucose levels were still measured in WT Pyr10 and *Trpc3^−/−^* mice as compared to WT. At T120, blood glucose was also higher in the WT Pyr10 and *Trpc3^−/−^* groups compared to the WT group (Figure 2a). Noteworthy, baseline blood glucose concentrations were lower in *Trpc3^−/−^* mice compared to WT (Figure 2a). During the entire IpGTT, there was a significant increase in plasma glucose in WT Pyr10 and *Trpc3^−/−^* versus WT as illustrated by the average curves (Figure 2b) and presented by the area under curve (AUC) measurements (Figure 2c). To note, blood glucose recorded in WT mice after glucose challenge, equaled almost 200 mg dl^−1^ which corresponds to 11 mm (Figure 2a,b).

At T0, all mice had comparable baseline plasma insulin. Plasma insulin was then lower at T15 in WT Pyr10 and *Trpc3^−/−^* mice compared to WT (Figure 2d). From T15 till T120, lower insulin levels were noted in WT Pyr10 and *Trpc3^−/−^* mice as compared to WT. At T120, plasma insulin for WT Pyr10 and *Trpc3^−/−^* persisted at lower levels as compared to WT (Figure 2d). The average curves and AUC measurements also supported these findings (Figure 2e,f). Overall in WT mice, plasma insulin presented ≈3‐fold increase after the glucose injection, from 0.6 to 1.76 ng mL^−1^, whereas in *Trpc3^−/−^* mice, ≈1.7‐fold plasma insulin increase was noted, from 0.66 to 1.12 ng mL^−1^.

Furthermore, in response to injection of arginine, plasma insulin at T15 was lower in WT Pyr10 and *Trpc3^−/−^* mice compared to WT (Figure 2g). From T15 till T120, lower insulin levels were noted in WT Pyr10 and *Trpc3^−/−^* mice as compared to WT. At T120, plasma insulin for WT Pyr10 and *Trpc3^−/−^* persisted at lower levels as compared to WT (Figure 2g). The average curves and AUC measurements showed the same patterns for the WT Pyr10 and *Trpc3^−/−^* groups as compared to WT (Figure 2h,i).

The next step was to conduct IpGTT using intraperitoneal (i.p.) injections of 4‐[[(1R,2R)‐2‐[(3R)‐3‐Amino‐1‐piperidinyl]‐2,3‐dihydro‐1H‐inden‐1‐yl]oxy]‐3‐chlorobenzonitrile dihydrochloride (SAR7334), a TRPC6‐specific inhibitor (5 mg kg^−1^).^[^ ^28^ ^]^ The dosage used was based on previously published data,^[^ ^28^ ^]^ resulting in a peak plasma concentration of less than 100 nm after 2 h of injection, effectively suppressing TRPC6 but having no impact on TRPC3 or TRPC7. Similar blood glucose and plasma insulin patterns were seen in both groups of mice during the whole IpGTT, as shown by the raw scatter plots of plasma glucose and insulin concentrations (Figure 2j,m), the average curves (Figure 2k,n), and the AUC values (Figure 2l,o).

### 2.3 Insulin Secretion in Human and Mouse Is Modulated by TRPC3

**Figure 3:**
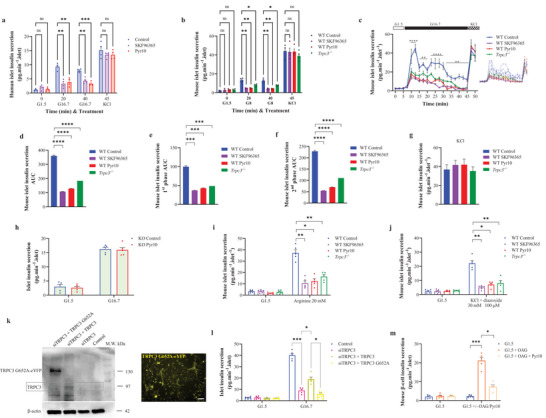
TRPC3‐dependent insulin secretion in human and mouse islets. a) Human islet static insulin secretion under low (G1.5) and high (G16.7) glucose, and KCl (30 mm). The human islets were treated with SKF96365 (30 µm) or Pyr10 (3 µm) 10 min prior to and until the end of the experiments. b) Raw scatter plots of insulin in perifused mouse islets under 8 mm glucose. c) Curve representation of insulin in perifused mouse islets, under the same conditions as in (a). On the right: curve representation of raw mouse islet insulin secretion data. d) AUC of the average mouse islet insulin secretion in (c). e) AUC of the average mouse islet insulin secretion during the first phase in (c). f) AUC of the average mouse islet insulin secretion during the second phase in (c). g) Average mouse islet insulin secretion during KCl addition in (c). h) Raw scatter plots of mouse islet static insulin secretion under glucose (G1.5) followed by high glucose (G16.7) in *Trpc3^−/−^* islets treated with Pyr10. i) Raw scatter plots of mouse islet static insulin secretion under glucose (G1.5) followed by arginine (20 mm). j) Raw scatter plots of mouse islet static insulin secretion under glucose (G1.5) followed by KCl (30 mm) + diazoxide (100 µm). k) Western blot showing the TRPC3 erase and replace experiment in isolated mouse beta cells, along with beta cells transfected with eYFP‐hTRPC3 G652A. l,m) Raw scatter plots of mouse beta‐cell static insulin secretion under the different settings. *n* = 200–300 islets from five independent experiments for each condition. *p < 0.05, **p < 0.01, ***p < 0.001, and ****p < 0.0001 versus control or WT control. Repeated measures two‐way ANOVA were used followed by Dunnett's tests in (a)–(c), (i), (j), and (m), Sidak's test in (h), or Tukey's test in (l). One‐way ANOVA were used followed by Tukey's tests in (d)–(g).

Human islets exhibited a significant increase in insulin secretion upon stimulation with high concentrations of glucose at 20 and 40 min (**Figure** **3**a). When human islets were treated with Pyr10 or 1‐[2‐(4‐Methoxyphenyl)‐2‐[3‐(4‐methoxyphenyl)propoxy]ethyl‐1H‐imidazole hydrochloride (SKF96365), significant reductions in insulin secretion were noted at 20 and 40 min (Figure 3a). However, Pyr10 and SKF96365 did not affect the insulin response induced by KCl (Figure 3a). Likewise, in mice and throughout both phases, insulin secretion of WT SKF96365, WT Pyr10, and *Trpc3^−/−^* islets was significantly lower than WT (Figure 3b–d). First, insulin secretion was stimulated by 8 mm glucose (Figure 3b), a concentration that mimics normal postprandial blood glucose (≈ 150 mg dL^−1^), and measured under static conditions. Subsequently, insulin secretion kinetics were studied in perifused islets under a dynamic stimulation index obtained with a glucose step from a low to a high 16.7 mm glucose as routinely performed in most literature ensuring robust insulin secretion to best represent islet functionality.^[^ ^3^, ^8^, ^10^, ^17^, ^29^, ^30^, ^31^, ^32^, ^33^, ^34^, ^35^, ^36^ ^]^ In WT control islets, the 8 mm glucose concentration induced an insulin increase of ≈5‐fold, from 2.588 to 13.455 pg min^−1^ islet^−1^ (Figure 3b), whereas the 16.7 mm glucose concentration induced an insulin increase of ≈18.5‐fold, from 2.425 to 44.783 pg min^−1^ islet^−1^ (Figure 3c). In *Trpc3^−/−^* islets, the insulin increase was approximately threefold from 2.934 to 8.493 pg min^−1^ islet^−1^ under 8 mm glucose (Figure 3b), and approximately sevenfold from 2.938 to 20.691 pg min^−1^ islet^−1^ under 16.7 mm glucose (Figure 3c). The effects of SKF96365 and Pyr10 were also illustrated by the individual AUC of the first phase (Figure 3e) and the second phase (Figure 3f). Upon KCl stimulation, mouse islets from all groups showed comparable increases in insulin secretion supported by comparable AUC (Figure 3g). The concentration of 3 µm Pyr10 was selected because it inhibits more than 75% of the channel activity while not affecting store‐operated calcium channels.^[^ ^24^ ^]^ To further validate the specificity of Pyr10, mouse *Trpc3^−/−^* islets were treated with Pyr10 under high glucose but no effects on static insulin secretion were observed (Figure 3h). Similar to Figure 3c, insulin secretion from *Trpc3^−/−^* islets presented a ≈5.5‐fold increase, from 2.97 to 16.213 pg min^−1^ islet^−1^ under 16.7 mm glucose (Figure 3h). The small fold difference might arise from the different islet batch preparations.

The in vitro insulin measurements for both WT and *Trpc3^−/−^* islets showed higher induction levels upon high glucose (16.7 mm corresponding to 300 mg dL^−1^) stimulation as compared to plasma insulin induction after glucose challenge in both mouse types (Figure 1d–f). Contrariwise, when lower glucose stimulation (8 mm) was used, insulin induction levels were comparable to the in vivo setting.

Under low glucose concentration and upon arginine addition, mouse WT cells showed a significantly higher islet insulin secretion than WT SKF96365, WT Pyr10, and *Trpc3^−/−^* (Figure 3i). Arginine promotes glucose‐induced insulin secretion^[^ ^37^ ^]^ via K_ATP_‐independent mechanisms such as membrane depolarization and activation of protein kinases A and C.^[^ ^34^ ^]^ Another common strategy for examining K_ATP_‐independent insulin secretion is the use of KCl and diazoxide concurrently.^[^ ^9^ ^]^ Diazoxide inhibits VDCCs by activating K_ATP_ channels, whilst KCl assures maximal membrane depolarization. Similarly, upon KCl + diazoxide addition, mouse islet insulin secretion showed a significantly lower increase in WT SKF96365, WT Pyr10, and *Trpc3^−/−^* compared to WT cells (Figure 3j).

In order to determine the mechanism by which glucose activates TRPC3, we focused on the lipid metabolite, diacylglycerol (DAG). Early theories suggested that DAG directly activates the TRPC3 channel.^[^ ^38^ ^]^ We took use of the recent discovery that a single mutation in TRPC3, G652A, blunts its ability to identify DAG^[^ ^39^ ^]^ in order to test the concept that DAG is related to TRPC3‐mediated insulin secretion in beta cells. In isolated mouse beta cells, knock‐down of TRPC3 resulted in the disappearance of the protein band at the expected molecular weight (97 kDa), and rescue with the G652A mutant form of TRPC3 increased the molecular weight, confirming the success of the erase and replace experiment (Figure 3k). Insulin secretion persisted when TRPC3 knock‐down was rescued by the WT form of TRPC3, but when the mutant form of TRPC3 was used for rescue, insulin secretion was severely affected (Figure 3l).

To further validate DAG action on TRPC3, the membrane‐permeable synthetic diacylglycerol analogue, 1‐Oleoyl‐2‐acetyl‐sn‐glycerol (OAG), was used on islets under low glucose conditions. Insulin secretion increased with OAG and was blunted under Pyr10 (Figure 3m).

### 2.4 *Trpc3* ^−/−^ Mice Are Glucose Intolerant but Not Diabetic

**Figure 4:**
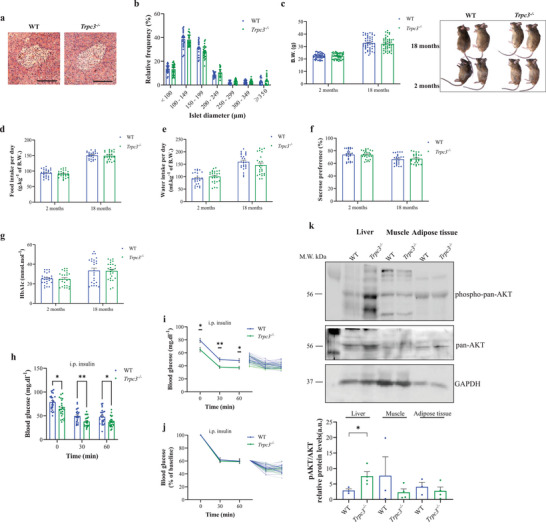
Metabolic Characterization of *Trpc3^−/−^* Mice. a) Histological images of Langerhans islets from WT and *Trpc3^−/−^* stained with H&E. Scale bars: 100 µm. b) Relative frequency of islet diameter in WT and *Trpc3^−/−^*. c–e) Body weight, and food and water intake of WT and *Trpc3^−/−^* mice at 2 and 18 months of age. On the right of (c), representative photographs of WT and *Trpc3^−/−^* mice. f) Sucrose preference of WT and *Trpc3^−/−^* mice at 2 and 18 months of age. g) HbA1c in WT and *Trpc3^−/−^* mice at 2 and 18 months of age. h) Raw scatter plots of blood glucose after insulin administration to WT and *Trpc3^−/−^* mice. i,j) Curve representation of blood glucose (w/o normalization) after insulin administration to WT and *Trpc3^−/−^* mice. On the right: curve representation of raw blood glucose data. k) Western blots and quantifications of protein kinase B (pan‐AKT) and phospho‐pan‐AKT (p‐AKT T308) in WT and *Trpc3^−/−^* mouse tissues (*n* = 3). *n* = 25–50 mice in each condition. *p < 0.05, **p < 0.01, and ****p < 0.0001 versus WT. Repeated measures two‐way ANOVA were used followed by Sidak's tests in (b)–(j). Unpaired two‐tailed *t*‐tests were used in (k).

Islets from WT and *Trpc3^−/−^* mice had similar histological features and comparable relative frequency for each diameter (**Figure** **4**a,b). At 2 and 18 months, there was no significant change in body weight between *Trpc3^−/−^* mice and WT mice (Figure 4c). In addition, both groups consumed equivalent amounts of food and water (Figure 4d,e) and there was no significant difference in sucrose preference (Figure 4f). At 2 and 18 months, there was no significant change in glycated hemoglobin (HbA1c) readings in percent between the two groups (Figure 4g).

During the insulin tolerance test, *Trpc3^−/−^* mice had significantly lower blood glucose levels than WT (Figure 4h,i). However, when blood glucose levels were adjusted to baseline, no significant changes were seen between the two groups (Figure 4j). Subsequently, the protein kinase‐B (AKT) pathway was evaluated in several tissues of *Trpc3^−/−^* and WT mice under fasting conditions as an attempt to explain the improved insulin sensitivity and given that the latter becomes comparable to WT when blood glucose levels are adjusted to fasting baseline. AKT phosphorylation is an essential step in the insulin signaling cascade, which leads to glucose absorption in target organs.^[^ ^40^ ^]^ *Trpc3^−/−^* animals had significant increases in phospho‐pan‐AKT and pan‐AKT expressions in the liver compared to WT mice (Figure 4k). On the other hand, in the muscle and adipose tissues, their expression remained unchanged. Besides, glyceraldehyde‐3‐phosphate dehydrogenase (GAPDH) expression remained constant within the same tissue type (Figure 4k).

Since the chow used in this study has a higher vitamin E content as compared to the Safe A04 diet (Table S2, Supporting Information), the impact of vitamin E food concentration on the amount of food consumed by the animals was investigated. First, mediobasal hypothalamus (MBH) neurons were isolated and treated with *α*‐tocopherol. Alpha‐tocopherol prevented the glucose‐excited neurons of WT mice from exhibiting a transient elevation in intracellular calcium in response to a rise in glucose level from 2.5 to 10 mmol L^−1^ (Figure S2a,b,i, Supporting Information). When the concentration of glucose was raised, reactive oxygen species (ROS) likewise increased, and alpha‐tocopherol also inhibited this response (Figure S2c,d,j, Supporting Information). However, *Trpc3^−/−^* animals showed a significant reduction in calcium and ROS rise in response to glucose stimulation. Intriguingly, alpha‐tocopherol had no discernible impact on both cell parameters in *Trpc3^−/−^* mice (Figure S2e–h,j, Supporting Information). Last, when vitamin E was administered to WT and *Trpc3^−/−^* mice, only the WT animals displayed increased food intake (Figure S2k, Supporting Information).

### 2.5 TRPC3 Is Not Required for Pancreatic Glucose Uptake

**Figure 5:**
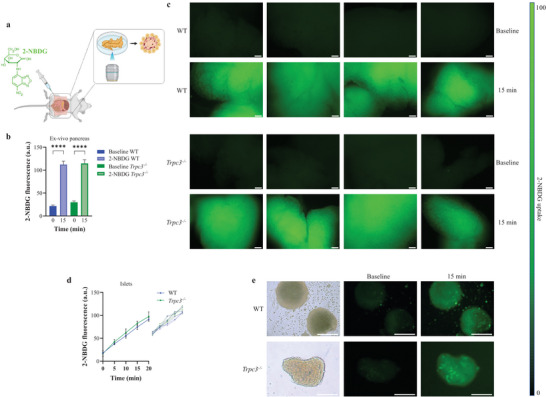
Pancreatic glucose uptake is independent of TRPC3. a) Schematic representation of ex vivo 2‐NBDG imaging (created in BioRender.com). b) Measurement of mouse WT and *Trpc3^−/−^* islet fluorescence at baseline and 15 min following 2‐NBDG administration in the ex vivo pancreas. *n* = 5 independent experiments for each condition. c) Representative 2‐NBDG fluorescence images of WT and *Trpc3^−/−^* pancreatic tissues. d) Measurements of 2‐NBDG fluorescence of isolated mouse islets from WT and *Trpc3^−/−^*. On the right: curve representation of raw 2‐NBDG fluorescence data in a.u. e) Representative fluorescence images of isolated islets from WT and *Trpc3^−/−^*. Each islet as a whole shows marked increase in fluorescence at 15 min. *n* = 200–300 islets from five independent experiments for each condition. Scale bars: 100 µm in (c) and (e). ****p < 0.0001 versus WT. One‐way ANOVA was used followed by Tukey's test in (b). Repeated measures two‐way ANOVA was used followed by Sidak's test in (d).

Since TRPC3 appeared to be involved in insulin secretion, we, nevertheless, sought to decipher whether this mechanism was indirectly mediated by TRPC3‐induced glucose sensing. The glucose fluorescent analogue, 2‐[*N*‐(7‐nitrobenz‐2‐oxa‐1,3‐diazol‐4‐yl) amino]‐2‐deoxy‐D‐glucose (2‐NBDG), was used for that purpose.^[^ ^41^, ^42^ ^]^ Mouse WT and *Trpc3^−/−^* islet fluorescence was evaluated in ex vivo pancreatic tissues at T0 and T15 following 2‐NBDG intramuscular injections (**Figure** **5**a). Both WT and *Trpc3^−/−^* mice displayed enhanced pancreatic fluorescence after receiving 2‐NBDG in the ex vivo setup (Figure 5b,c). Concordantly, the increase in fluorescence in WT and *Trpc3^−/−^* isolated islets treated in vitro with 2‐NBDG was comparable over time (Figure 5d,e). To note, intracellular Ca^2+^ content influences glucose absorption in other cell types such as intestinal cells;^[^ ^43^ ^]^ consequently, TRPC3 channel involvement in glucose absorption may be tissue‐specific and not that prominent in beta cells.

### 2.6 TRPC3 Mediates Glucose‐Stimulated Calcium Oscillations

**Figure 6:**
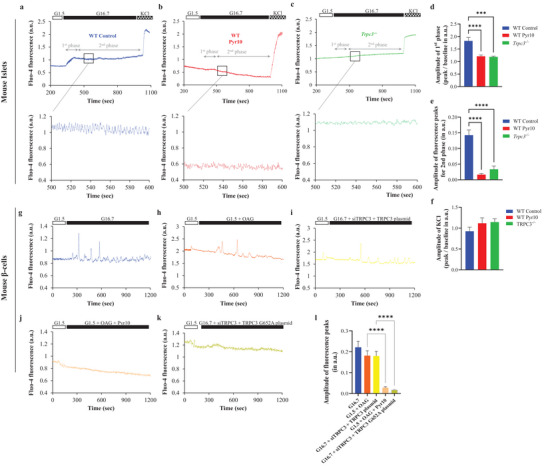
Glucose‐stimulated calcium oscillations are mediated by TRPC3. a–c) Representative fluo‐4 calcium signals, in isolated mouse islets, exposed to low (G.15) and high (G16.7) glucose, and KCl (30 mm). The WT islets were treated with Pyr10 (3 µm) 10 min prior to and until the end of the experiments. The bottom graphs in each panel represent a small fraction of the second phase with the characteristic calcium oscillations. d) Average increase in fluorescence ratio after stimulation with 16.7 mm glucose w/o Pyr10 in islets from WT and *Trpc3^−/−^* mice. e) Amplitude of fluorescence peaks in individual experiments from WT and *Trpc3^−/−^* islets, measured in a fraction of the second phase as shown in (a)–(c). f) Average increase in fluorescence ratio after stimulation with KCl. ***p < 0.001, and ****p < 0.0001 versus WT Control. g–k) Representative fluo‐4 calcium signals, in isolated mouse beta cells, exposed to low (G1.5) or high glucose (G16.7), OAG w/o Pyr10, and subjected to knock‐down and rescue experiments with siTRPC3 and either a wild‐type TRPC3 or a mutant form, TRPC3 G652A. l) Amplitude of fluorescence peaks in mouse beta cells under the different settings. ****p < 0.0001 versus G1.5 + OAG and G16.7 + siTRPC3 + TRPC3 plasmid. *n* = 70–90 islet cells from three independent experiments for each condition. One‐way ANOVA were used followed by Tukey's tests in (d), (e), and (k).

Glucose‐stimulated Ca^2+^ (GSCa) was studied in mouse WT and *Trpc3^−/−^* islets at various glucose concentrations (**Figure** **6**). Throughout the whole 20‐min procedure, substantially greater islet calcium levels were observed in WT islets compared to WT Pyr10, and *Trpc3^−/−^* islets (Figure 6a–c). The first phase was inhibited in WT Pyr10 and *Trpc3^−/−^*, which was validated by the fluorescence amplitude measurements (Figure 6d). Besides, high glucose concentration evoked, in both WT and *Trpc3^−/−^* islets, a characteristic oscillatory rise in calcium that lasted for several minutes. Strikingly, calcium peaks during the oscillations in WT Pyr10 and *Trpc3^−/−^* islets were significantly lower in amplitude throughout the course of the experiments (Figure 6b–e). Mouse islets from all groups showed equivalent increases in calcium levels upon maximal depolarization with KCl (Figure 6f). When beta cells were dispersed from mouse islets and cultured, they exhibited calcium oscillations in response to high glucose (Figure 6g,l). These oscillations were replicated with the diacylglycerol analogue, OAG, under low glucose conditions (Figure 6h,l). Knock‐down of endogenous TRPC3 was then conducted by siTRPC3 and rescue was done by a plasmid carrying the WT form of TRPC3; under this condition, calcium oscillations subsisted (Figure 6i,l). However, when Pyr10 was used with OAG, and when the mutant form of TRPC3, G652A, was used for rescue, calcium oscillations were abrogated (Figure 6j–l).

### 2.7 TRPC3 Activation by a Small‐Molecule Activator Stimulates Insulin Secretion

TRPC3 was activated with 1,3‐Dihydro‐1‐[1‐[(5,6,7,8‐tetrahydro‐4H‐cyclohepta[b]thien‐2‐yl)carbonyl]‐4‐piperidinyl]‐2H‐benzimidazol‐2‐one (GSK1702934A), a fairly specific TRPC3 activator.^[^ ^44^ ^]^ GSK1702934A can also activate TRPC6;^[^ ^45^ ^]^ however, the required concentration has to be greater than 1 µm, which is more than ten times the dose employed in our study (80 nm). As a result, no effect on insulin secretion was observed under high glucose stimulation.

**Figure 7:**
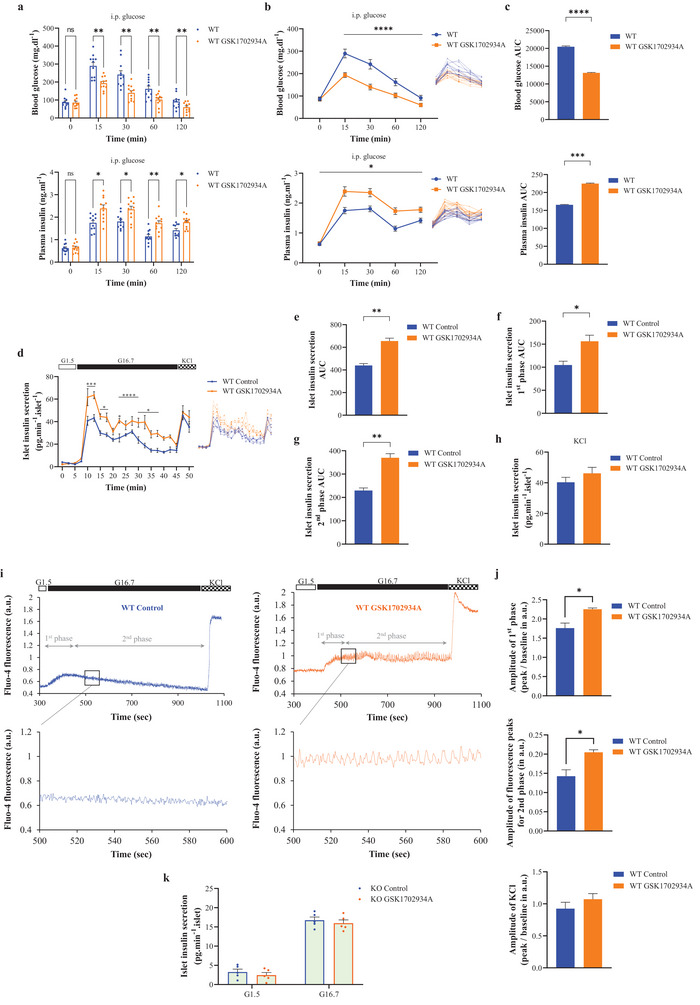
Pharmacologic modulation of TRPC3 by a small‐molecule activator stimulates insulin secretion. a) Raw scatter plots of plasma glucose and insulin concentrations during IpGTT. b) Curve representation of the average plasma glucose and insulin concentrations during IpGTT. On the right: curve representation of raw plasma glucose and insulin concentration data. c) AUC of the average plasma glucose and insulin concentrations in (b). d) Curve representation of insulin secretion in mouse perifused islets, exposed to low (G1.5) and high (G16.7) glucose, and KCl (30 mm). *n* = 200–300 islets from five independent experiments for each condition. WT islets were treated with GSK1702934A (80 nm) 10 min prior to and until the end of the experiments. On the right: curve representation of raw islet insulin secretion data. e–g) AUC of the total, first, and second phases of islet insulin secretion in (d). h) Average islet insulin secretion during the KCl addition in (d). i) Representative fluo‐4 calcium signals and oscillations, in isolated mouse islets, exposed to low (G.15) and high (G16.7) glucose, and KCl (30 mm). *n* = 30–90 islet cells from three independent experiments for each condition. j) Average increase in fluorescence ratio and amplitude of fluorescence peaks during the oscillations after stimulation with 16.7 mm glucose or KCl w/o GSK1702934A in islets from WT mice. *p < 0.05 WT GSK1702934A versus WT Control. k) Raw scatter plots of islet static insulin secretion under glucose (G1.5) followed by high glucose (G16.7) in *Trpc3^−/−^* islets treated with GSK1702934A. Repeated measures two‐way ANOVA were used followed by Sidak's tests in (a), (b), (d), and (k). One‐way ANOVA was used followed by Tukey's test in (c). Unpaired two‐tailed *t*‐tests were used in (e)–(h) and (j).

During IpGTT, blood glucose was lower in WT GSK1702934A compared to WT at T15 (**Figure** **7**a,b). At T120, blood glucose for the WT GSK1702934A group persisted at lower values compared to WT (Figure 7a,b). The AUC measurements supported these findings (Figure 7c). GSK1702934A acute treatment increased plasma insulin at T15 compared to control mice (Figure 7a,b). At T120, plasma insulin was also higher after GSK1702934A (Figure 7a,b). These results were further illustrated by the AUC measurements (Figure 7c).

In isolated mouse islets, GSK1702934A treatment led to a higher insulin secretion upon glucose stimulation, throughout both phases (Figure 7d). Upon KCl addition, islets from all groups showed a comparable increase in insulin secretion. These results were further supported by comparable AUC values (Figure 7e–h).

In the GSCa evaluation, higher islet Ca^2+^ under GSK1702934A was noted upon glucose stimulation (Figure 7i,j). This was supported by the amplitude of calcium peaks during the oscillations (Figure 7j). Most importantly, GSK1702934A induced a higher amplitude of calcium oscillations (Figure 7j). Upon KCl addition, islets from all groups showed a comparable increase in Ca^2+^ levels (Figure 7i,j). To further confirm GSK1702934A specificity at low concentrations, it was tested on *Trpc3^−/−^* islets under high glucose but did not have any effect on insulin secretion (Figure 7k).

### 2.8 TRPC3 Activation Alleviates Type 2 Diabetes Hallmarks in Mice

**Figure 8:**
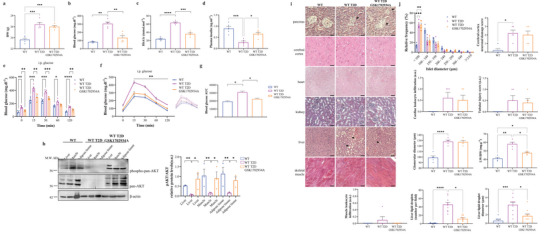
Pharmacologic modulation of TRPC3 by a small‐molecule activator ameliorates experimental type 2 diabetes. a–d) Body weight, fasting blood glucose, HbA1c, and plasma insulin in wild‐type (WT) control and diabetic mice (T2D) w/o GSK1702934A. e,f) Raw scatter plots and curve representation of the average of plasma glucose concentrations during IpGTT in the different animal groups. On the right in (e): curve representation of raw plasma glucose concentration data. g) Area under curve (AUC) of the average plasma glucose concentrations in (e) and (f). h) Western blots and quantifications of protein kinase B (pan‐AKT) and phospho‐pan‐AKT (p‐AKT T308) in WT control and T2D mice w/o GSK1702934A (*n* = 3). i) Representative organ sections stained with H&E from WT control and T2D mice w/o GSK1702934A. j) Histological analysis of mice tissues and liver weights in the different animal groups. *n* = 5 mice for each group. *p < 0.05, **p < 0.01, ***p < 0.001, and ****p < 0.0001 versus WT T2D. Brown–Forsythe and Welch ANOVA were used followed by Dunnett's T3 tests in (a)–(c). One‐way ANOVA were used followed by Tukey's tests in (d), (g), and (h). Repeated measures two‐way ANOVA were used followed by Sidak's tests in (e) and (f). Kruskal–Wallis one‐way ANOVA on ranks or ordinary one‐way ANOVA were used followed by either Dunn's or Tukey's multiple comparisons tests in (j).

TRPC3 activation by small‐molecule GSK1702934A was evaluated in diabetic mice (T2D). T2D mice had increased body weight, however, GSK1702934A had no effect on this parameter (**Figure** **8**a). Fasting blood glucose increased in T2D mice and was subsequently lower in GSK1702934A treated animals at 4 weeks compared to untreated ones (Figure 8b). Similarly, HbA1c was lower in T2D GSK1702934A mice at 4 weeks compared to T2D untreated ones (Figure 8c). On the other hand, plasma insulin declined in T2D mice whereas GSK1702934A increased its levels (Figure 8d). During IpGTT, a significant increase in plasma glucose was noted in T2D mice throughout the test period as compared to non‐diabetic animals (Figure 8e–g). Conversely, GSK1702934A treatment ameliorated glucose tolerance in T2D mice (Figure 8e–g). Similarly, AKT phosphorylation was significantly abrogated in tissues of T2D mice, whilst GSK1702934A restored the AKT activation status in these tissues (Figure 8h). Next, the potential adverse effects of GSK1702934A were then assessed histologically on a variety of mouse tissues (Figure 8i,j). Langerhans islets were smaller and less numerous in T2D mice, which is a classic sign in this high‐fat streptozotocin (STZ) diabetic model. T2D mice had also increased cerebral neuronal damage, glomerular diameter, and tubular injury, with signs of cardiac inflammation. Besides, the liver presented obvious evidence of fat deposition, with increased tissue weight, lipid droplet presence, and size. In contrast, GSK1702934A did not appear to impact neither the morphology of the islets nor the cerebral cortex, heart, kidney, and skeletal muscle structures. However, GSK1702934A therapy caused a reduction in liver weight, with a clear effect on the fatty liver phenotype (Figure 8i,j).

## 3 Discussion

The current work identifies TRPC3 as an insulin secretion regulator in human and mouse pancreatic beta cells. TRPC3 is suggested to ultimately control GSCa as well as insulin secretion. The most striking aspect of our findings is that the pharmacological activation of TRPC3 alleviates diabetes hallmarks in treated animals (Figure S3, Supporting Information). These insights into pancreatic TRPC3 function are considered as an essential step toward a better understanding of the intricate insulin secretion physiology and pave the way for the development of promising diabetes‐focused treatments.

Among TRP channels, transient receptor potential melastatin TRPM2, 3, 4, and 5, and canonical TRPC1, have so far been recognized as involved in the control of insulin secretion.^[^ ^8^, ^9^, ^31^, ^32^, ^46^, ^47^ ^]^ Other TRPs are reportedly present in beta cells though their function is not yet been elucidated.^[^ ^19^ ^]^ TRPC3 channels can be found in the pancreas exocrine acini^[^ ^15^ ^]^ but they remain of unrecognized functional role in the endocrine tissue with contradictory literature hovering around their existence in beta cells.^[^ ^16^, ^17^, ^19^, ^20^, ^21^ ^]^ To the best of our knowledge, this is the first report to demonstrate TRPC3 protein expression at a low level and functional relevance in islets from human and mouse pancreatic tissues. TRPC3 was weakly detected in beta cell regions, where insulin and the GLUT2 transporter are also present. Additionally, TRPC3 expression was dispersed across the human pancreatic islet, which correlates to the distribution of alpha cells. However, it was located near the periphery of the mouse's islets, similar to how alpha cells are distributed in this species.^[^ ^48^ ^]^ TRPC3 may thus have a role in glucagon secretion since the latter is also calcium‐dependent.^[^ ^49^ ^]^ These results are in line with previous animal studies that showed the functional relevance of TRPC3 in rodent beta cells.^[^ ^16^, ^17^ ^]^ They converge to a certain extent with previous reports where RNA‐seq demonstrated little^[^ ^18^ ^]^ to no presence of TRPC3 in human islets^[^ ^19^, ^20^, ^21^ ^]^ due to genuine TRPC3 expression variations between humans and rats or the very low level of TRPC3 expression in human beta cells^[^ ^48^ ^]^ that cannot be properly identified with the number of reads acquired in the tests. At last, using two TRPC3 antibodies, the existence of the protein at a low level was further confirmed in human and mouse islets.

Even though numerous TRP channels are expressed in beta cells, only a few have been connected to insulin secretion,^[^ ^19^ ^]^ and even less have been shown to have functional significance in islets in vivo.^[^ ^7^, ^8^, ^31^, ^33^ ^]^ Pharmacological inhibition of TRPC3 but not TRPC6, and its deletion in mice, resulted in glucose intolerance and defective insulin secretion, with substantially lower plasma insulin levels in both the first and second phases of insulin secretion. Based on these findings, TRPC3 appears to have a critical role in insulin secretion.

To fully understand TRPC3's involvement, we isolated human and mouse islets and evaluated insulin secretion in vitro under various settings. Under high glucose, human and WT mice islets treated with SKF96365 or Pyr10, as well as *Trpc3^−/−^* islets, secreted considerably less insulin than WT islets. The insulin induction index was higher in vitro than in vivo, which might be explained by the higher glucose concentration used in vitro. Indeed, it has been shown that 16.7 mm glucose generates greater insulin bursts than 11 mm ^[^ ^17^, ^32^, ^35^, ^36^, ^50^ ^]^ which is regarded as the half‐maximal insulin release stimulating concentration. Furthermore, such increased in vitro insulin secretion induction indices are often reported when compared to in vivo.^[^ ^21^, ^51^ ^]^

TRPC3 appears to accomplish the primary function in insulin secretion since there was no additive impact of the non‐selective TRPCs inhibitor, SKF96365, compared to Pyr10. However, prior research found that TRPC3 was present in rat islets and INS‐1E cell line but the TRPC1 and ORAI1 channels were the predominant K_ATP_‐independent channels, ruling out TRPC3 as a candidate based on TRPC3 blockage with Pyr3 and knockdown by a dominant‐negative expression.^[^ ^10^ ^]^ These findings are intriguing since Pyr3 inhibits both TRPC3 and ORAI1.^[^ ^24^ ^]^ Besides, in the GSIS experiment, the paper employed the INS‐E1 cell line rather than primary cells, which might explain the discrepancy.

The influence on insulin secretion was found in both the first and the second phases of insulin secretion in our settings. The first phase initiates with a glucose‐induced increase in the ATP/ADP ratio, which blocks K_ATP_ and opens voltage‐gated Na^+^ and Ca^2+^ channels. Some investigations postulated that TRP channels had a function in the first phase by depolarizing VDCCs,^[^ ^4^, ^7^ ^]^ which might explain TRPC3 inhibition with Pyr10 and *Trpc3^−/−^* impacts on insulin secretion during the first phase. Therefore, investigating the functional interaction between TRPC3 and VDCCs in beta cells is warranted. Conversely, the second phase is dependent of the involvement of numerous ion channels, resulting in calcium oscillations,^[^ ^1^, ^4^ ^]^ which aligns with the effects of TRPC3 inhibition on the second phase of insulin secretion. Furthermore, when measuring maximal insulin secretion that reflects insulin storage, adding KCl resulted in a similar rise in insulin. As a result, TRPC3 inhibition and deletion seem to have little impact on insulin reserve and its storage in the cell.

We then sought to validate TRPC3's involvement in K_ATP_‐independent pathways. In comparison to WT mice, plasma insulin stimulated by arginine was inhibited by Pyr10 treatment and in *Trpc3^−/−^* animals. Furthermore, in vitro arginine supplementation of islets inhibited insulin secretion in SKF96365, Pyr10, and *Trpc3^−/−^* islets. Similarly, when KCl and diazoxide were added, islet insulin secretion increased much less in WT SKF96365, WT Pyr10, and *Trpc3^−/−^* cells than WT cells. These findings are undeniably in favor of TRPC3 involvement in K_ATP_‐independent pathways.

As *Trpc3^−/−^* mice have defective insulin secretion, Langerhans islets were consequently examined to check whether they were less prevalent, atrophied, or hypertrophied. Histological data of Langerhans islets from WT and *Trpc3^−/−^* pancreatic tissues were comparable, as was the relative frequency of islets in the two groups. After that, the metabolic characterization revealed that *Trpc3^−/−^* mice are not diabetic even at 18 months of age, prompting us to investigate the peripheral response to insulin as a potential compensation mechanism. When controlled for baseline glucose, there was no substantial fluctuation in blood glucose concentrations, indicating a normal postprandial response to insulin but a higher sensitivity at baseline. *Trpc3^−/−^* animals showed increased AKT expression and activity representing a compensatory mechanism in the hepatic AKT pathway, which might explain the *Trpc3^−/−^* mice's quasi‐normal phenotype as well as their low fasting blood glucose. Indeed, TRPCs have been related to the PI3K‐Akt signaling pathways in a variety of cell types with the presence of a putative loop between TRPCs and PI3K‐AKT, where TRPCs activate PI3K‐AKT and the latter reactivate TRPCs.^[^ ^52^, ^53^ ^]^ Additionally, similar findings were previously described whereby AKT activation was increased in *Trpc3/6/7* triple knockout mouse AKT.^[^ ^13^ ^]^

An important study by Chretien et al.^[^ ^54^ ^]^ observed similar glucose intolerance in *Trpc3^−/−^* mice due to their aberrant TRPC3‐dependent hypothalamic glucose sensing. However, the mice consumed more food and had a larger bodyweight in their experimental conditions than WT mice, accompanied by higher fasting plasma glucose levels. The main possible confounding factor that might explain the disparity in food intake is the rodent diet composition. Mice are extremely susceptible to changes in their nutritional status, with even slight dietary adjustments eliciting profound physiological changes.^[^ ^55^ ^]^ The chow used herein is richer in protein, zinc, vitamin A, and vitamin E (Table S2, Supporting Information), all of which are known to alter body weight.^[^ ^56^, ^57^, ^58^, ^59^, ^60^, ^61^, ^62^ ^]^ TRPC3 channels are essential for the response of mediobasal hypothalamic glucose‐excited neurons to glucose, as well as the central influence of glucose on insulin production and food intake.^[^ ^54^, ^63^ ^]^ This TRPC3 activity is mediated by a mechanism that is reliant on the production of reactive oxygen species (ROS/H_2_O_2_), which may be efficiently prevented by antioxidants like Trolox and catalase. We hypothesized that the richer content of vitamin E in the mouse chow used in our work may hinder ROS‐mediated hypothalamic TRPC3 glucose sensing in WT mice, rendering them equivalent to *Trpc3^−/−^* animals in terms of food intake. To test this theory, MBH neurons from WT and *Trpc3^−/−^* mice were first cultured and glucose‐induced calcium and ROS oscillations were investigated in glucose‐excited neurons w/o *α*‐tocopherol treatment. Calcium and ROS responses in WT neurons exclusively were abolished under *α*‐tocopherol resembling the ones exhibited in *Trpc3^−/−^* cells. In line with these results, when WT and *Trpc3^−/−^* mice were supplemented with vitamin E, food intake only increased in WT animals, corroborating the previous hypothesis (Figure S2, Supporting Information).

Furthermore, Chretien et al. reported no peripheral insulin sensitivity compensation in *Trpc3^−/−^* mice. Nonetheless, they measured AKT activation following insulin administration, as opposed to our work, where we investigated the AKT status in fasting animal tissues. These findings do not diverge but rather point to a convergent mechanism in which *Trpc3^−/−^* mice peripheral tissues have a typical postprandial insulin action but a greater sensitivity during fasting; this phenotype is consistent with glucose intolerance observed in prediabetes but does not yet fit overt diabetes.^[^ ^64^ ^]^ These observations are complemented by the identical insulin sensitivity test curves obtained in our study between WT and *Trpc3^−/−^* mice after adjusting blood glucose levels to baseline. Finally, during the oral glucose tolerance test (OGTT), Chretien et al. discovered that mice had equal plasma insulin levels. In contrast to the IpGTT employed herein, which does not involve GLP1, the OGTT test is known to activate GLP1, potentially obscuring phenotypic differences between WT and *Trpc3^−/−^* mice. In addition, OGTT may trigger interference with other channels, such as TRPM4 and TRPM5.^[^ ^47^ ^]^

GSIS is primarily dependent on Ca^2+^ entry via VDCCs. Because pancreatic beta cells without K_ATP_ display calcium signals caused by glucose stimulation, calcium oscillations of the second phase are referred as K_ATP_‐independent pathways in GSIS.^[^ ^65^ ^]^ Since TRPC3 was not linked to glucose sensing, we decided to investigate its involvement in Ca^2+^‐dependent oscillations. The initial phase of the islet response to glucose was marked by a first surge in intracellular Ca^2+^, followed by a plateau phase marked by high‐frequency Ca^2+^ oscillations reflecting the typical islet response to glucose.^[^ ^30^, ^35^, ^66^ ^]^ When TRPC3 activity was pharmacologically or genetically abolished, islets showed a reduced calcium rise throughout the first phase. In addition, calcium oscillations had smaller amplitudes during the second phase. A number of studies have shown that closure of the K_ATP_ channels alone is insufficient to achieve the degree of depolarization needed for effective activation of the VDCCs, indicating that the participation of additional components altering the membrane potential is necessary. Accordingly, TRP channels have been proposed to provide sufficient depolarization to open VDCCs in islets and other cells.^[^ ^10^, ^67^ ^]^ Therefore, TRPC3 may also contribute to insulin secretion via its impact on VDCCs activity, which would explain its influence on both stages of insulin release.

Noteworthy, the reduced glucose‐induced Ca^2+^ signals with TRPC3 inhibition or knockout to the extent of abolition in some islets might seem discordant with the presence of GSIS, despite reduced, in the islets. Apart from the common consensus that K_ATP_‐dependent pathways drive insulin secretion, considerable GSIS also occurs when K_ATP_ channel closure is inhibited, highlighting the importance of K_ATP_ channel‐independent pathways in insulin release.^[^ ^65^, ^68^ ^]^ Although K_ATP_‐dependent pathways are particularly important in the first phase of insulin secretion, and ATP may be involved in the second phase, it is clear that other factors are also involved via the so‐called metabolic amplification pathway, which is likely independent of the triggering calcium‐dependent pathway.^[^ ^69^ ^]^ Among ≈60 metabolites, suggested stimulus and secretion coupling factors include NADPH, GTP, the glycerolipid/free fatty acid (FFA) cycle, monoacylglycerol, and glutamate,^[^ ^70^, ^71^ ^]^ which may be implicated in both stages of insulin secretion.^[^ ^72^ ^]^ Furthermore, glucose may stimulate insulin secretion by the islets, even under stringently Ca^2+^‐free conditions, given pre‐exposure of the islets to activators of PKA and PKC.^[^ ^73^, ^74^, ^75^ ^]^ In addition to glucose, L‐leucine and L‐arginine can also increase insulin secretion in strictly Ca^2+^‐free conditions through a cAMP‐dependent mechanism.^[^ ^76^, ^77^ ^]^ Hence, the metabolic amplification pathway might be responsible for the remaining GSIS with TRPC3 inhibition in our study, despite the important blockade of intracellular calcium.

The TRPC3 channel's transmembrane region has a DAG‐sensing structure, as subsequently shown by Lichtenegger et al.^[^ ^39^ ^]^ An investigation of the TRPC3 channel's structure by single particle cryo‐EM supported these results.^[^ ^78^ ^]^ In more recent research, it has been shown that DAG has the capacity to engage with certain lipid‐interaction sites on TRPC3 and to produce a sensitized closed state, which facilitates rapid and effective channel opening in response to ensuing activating stimuli.^[^ ^79^ ^]^ In beta cells, DAG is present and amplifies glucose‐dependent insulin release through the malonyl‐CoA pathway.^[^ ^80^ ^]^ Pyruvate carboxylase facilitates beta‐cell glucose metabolism to create citrate, which is exported from mitochondria and converted to malonyl‐CoA. Malonyl‐CoA production connects glucose metabolism to increased cytosolic FFAs since it inhibits mitochondrial FFA oxidation. Increased FFA availability promotes long‐chain fatty acyl‐CoA esters and DAG production. The latter metabolite stimulates insulin secretion by protein kinase C‐mediated phosphorylation of secretory granule‐associated proteins. Calcium‐dependent exocytosis is therefore amplified.^[^ ^80^, ^81^ ^]^ Furthermore, we evaluated the beta‐cells' ability to produce calcium oscillations and release insulin in response to glucose stimulation after transfecting them with the G652A‐TRPC3 mutant. The glycine residue G652, harbored in a fenestrated subunit interface close to the TRPC3 lipid selectivity filter, is critical for molecular recognition and accommodation of lipid mediators by the channel as well as for enabling flexibility for gating motions within the pore domain. Mutating the glycine residue to alanine leads to impaired basal and phospholipase C‐stimulated TRPC3 channel functions. In this G652A mutation, lipid‐dependent gating is modified with altered threshold for lipid regulation, whereas the TRPC3 open pore characteristics remain unaffected. The fact that the G652A mutation resulted in the removal of activation by direct bath administration of 1‐Stearoyl‐2‐Arachidonoyl‐*sn*‐Glycerol, a very potent agonist of lipid DAG‐regulated TRPC channels, lends more credence to G652's crucial involvement in lipid sensitivity.^[^ ^39^ ^]^ Our findings showed that G652A TRPC3‐transfected cells lacked calcium oscillations and had a diminished ability to secrete insulin. At last, OAG‐induced Ca^2+^ oscillations disappeared when TRPC3 was blocked, and insulin secretion followed a similar pattern. Overall, we demonstrate that beta‐cell DAG controls TRPC3‐mediated insulin secretion.

The question now is how does this information translate into the clinical setting? Calcium oscillations are disrupted in diabetic islets, thus deeming them a promising therapeutic target to investigate.^[^ ^82^, ^83^ ^]^ The impact of TRPC3 activation in mice was then evaluated. In vivo, upon GSK1702934A acute TRPC3 activation, glucose tolerance was improved concordantly with significant plasma insulin elevation. These results correlated with an increased insulin secretion in vitro in response to GSK1702934A at high glucose concentrations. Consistently, GSCa oscillations were clearly amplified with GSK1702934A.

Ultimately, chronic constitutive TRPC3 activation by GSK1702934A was associated with significant amelioration of the type 2 diabetic phenotype in mice as assessed by fasting blood glucose and HbA1c levels. Fasting insulin levels were also improved under GSK1702934A as well as glucose tolerance assessed by IpGTT. AKT activation was severely affected in the liver, muscles, and adipose tissue of diabetic mice, and TRPC3 activation reverted AKT phosphorylation to normal. According to tissue histological analyses, GSK1702934A did not show any significant hazardous flags in vivo and had no observable adverse effects on the animals. The TRPC3‐mediated insulin secretion hypothesis may thus be tested in experimental models using GSK1702934A as a suitable small‐molecule. The decreased liver weight and fat buildup in the hepatic tissue of T2D mice served as the final piece of evidence for the possible therapeutic effect of TRPC3 activation.

TRPC3 has been implicated in the regulation of metabolism in a variety of tissues, including the central nervous system and skeletal muscles, as previously stated. TRPC3 deletion in different tissue types may potentially impact our in vivo animal research utilizing a global *Trpc3^−/−^* mouse. Whilst endocrine and exocrine pancreatic compartments are thought to be functionally separate, there are direct and indirect links between them that promote cellular development, differentiation, survival, and function throughout normal physiological processes as well as during illness.^[^ ^84^ ^]^ For example, inhibiting pancreatic elastase, which is generated by exocrine acinar cells, has been demonstrated to promote beta cell proliferation.^[^ ^85^ ^]^ Furthermore, duct cells release a variety of growth factors, cytokines, and eicosanoids that may have a variety of subtle effects on islet cells.^[^ ^86^ ^]^ Because TRPC3 deletion impacts Ca^2+^ signaling and agonist‐stimulated acinar secretion,^[^ ^87^, ^88^ ^]^ it may also deregulate acinar/duct function, impairing endocrine islets. Although this is a limitation to our investigation, the majority of our experiments relied on in vitro data from mouse and human isolated islets. This approach is considered as a cornerstone in insulin secretion studies, since the isolated islets are put in a well‐controlled environment completely devoid of peripheral tissue interference. Consequently, the results obtained from isolated islets corroborated the metabolic phenotype seen in the knockout mice. Nonetheless, studying a beta‐cell specific *Trpc3^−/−^* mouse could be also of valuable interest. Moreover, our results and those in literature ascertain the co‐existence of both voltage‐dependent and voltage‐independent Ca^2+^ influxes in beta cells, coordinating the intricate insulin secretion mechanism. Some unanswered questions still remain regarding the mechanism by which TRPC3 activity regulates VDCC activity during the first insulin secretion phase, which merit further investigation.

In conclusion, TRPC3 is found at a low level in both human and mouse Langerhans islets and appears to exhibit a critical function in the GSIS pathway. Our findings prepare the ground for the development of novel drugs for the treatment of diabetes.

## 4 Experimental Section

Additional detailed methods are available in Supporting Information.

### Animals

The study was carried out on male TRPC3 knockout (*Trpc3^−/−^*) mice aged 2 and 18 months with their age‐matched littermate WT controls. Mice were developed at the Comparative Medicine Branch of the NIEHS on a 129SvEv/C57BL/6J mixed background as previously described.^[^ ^89^ ^]^ The in vivo and in vitro protocols on animals and human tissues are represented in the flowchart of Figure S1, Supporting Information.

### Intraperitoneal Glucose Tolerance Tests and Arginine Challenge

The IpGTT was conducted instead of the OGTT because of its ease of use, while also being less stressful for animals than the intragastric gavage technique during OGTT. Add to that, it circumvented the incretin response that might amplify GSIS^[^ ^90^, ^91^ ^]^ and activate TRPM4/5 channels.^[^ ^47^ ^]^ IpGTT was first carried out on three groups of 25 mice per group (three independent experiments): WT, WT with Pyr10 (specific TRPC3 inhibitor),^[^ ^24^ ^]^ and *Trpc3^−/−^* mice. Pyr10 (Sigma‐Aldrich, St. Louis, MO, USA) was acutely administered (i.p.) 10 min prior to the IpGTT, at a dose of 8 µg kg^−1^ by analogy to previously described daily doses.^[^ ^14^, ^92^ ^]^ For TRPC3 activation testing, two additional mouse groups were used: WT (*n* = 11) and WT acutely administered (i.p.) 10 min before IpGTT with a specific TRPC3 small‐molecule activator GSK1702934A^[^ ^44^ ^]^ (kindly provided by Pr. Klaus Groschner, Gottfried‐Schatz‐Research‐Centre‐Biophysics, Medical University of Graz, Austria) at a dose of 35 µg kg^−1^ (*n* = 11). Similarly, insulin secretion was assessed following an i.p. injection of arginine (1 g kg^−1^) in another set of mice: WT (*n* = 25), WT previously administered with Pyr10 (*n* = 25), and *Trpc3^−/−^* mice (*n* = 25) (three independent experiments). For TRPC6 inhibition experiments, SAR7334 (Tocris Bioscience, Bristol, UK) was used at a dose of 5 mg kg^−1^ in vivo (i.p.)^[^ ^28^ ^]^ and two groups of mice (*n* = 6 each) were studied, WT and WT SAR7334. Plasma insulin was measured according to Crystal Chem's Ultra‐Sensitive Mouse Insulin ELISA Kit (IL, USA).

### Human and Animal Islet and Beta‐Cell Isolation, Treatment, and Transfection Protocols

Animals were anesthetized by a mixture of ketamine (75 mg kg^−1^; Interchemie, Waalre, Holland) and xylazine (10 mg kg^−1^; RotexMedica, Trittau, Germany). Islets were then isolated as previously described^[^ ^93^ ^]^ using collagenase A (Roche Diagnostics, Germany) with a Percoll gradient. Islets were further dispersed with 0.25% trypsin for 10 min at 37 °C to isolate beta cells for the siRNA/plasmids transfection experiments, and related insulin and intracellular calcium measurements.

Human pancreatic samples were collected from patients (*n* = 4) undergoing the Whipple Procedure for pancreatic tumor resection. Islets were isolated similarly as for mice with an additional hand‐picking step, and tissues were also used for immunofluorescence studies.

WT islets were treated with Pyr10 (3 µm), a non‐specific Ca^2+^ entry blocker SKF96365 (30 µm) (Sigma‐Aldrich, St. Louis, MO, USA), or GSK1702934A (80 nm) for 10 min prior to starting the perifusion experiments and till the end. A final KCl (30 mm) infusion was used to completely depolarize the islets and check for total insulin content. For arginine (20 mm) and KCl (30 mm) + diazoxide (100 µm) testing, static insulin secretion was studied for 20 min. Isolated beta cells were treated with OAG (100 µm) (Cayman Chemical, Ann Arbor, MI, USA) as a surrogate for diacylglycerol.

Erase and replace (knock‐down and rescue) experiments in beta cells were conducted using Attractene transfection (Qiagen, Hilden, Germany) and siRNA targeting to TRPC3. Cells were allowed to recover for 48 h before re‐transfecting with a plasmid encoding either the WT form of TRPC3 or a mutant form (G652A). hTRPC3 plasmid was from Origene (Rockville, Maryland, USA). hTRPC3 was also mutagenized within the transmembrane pore domain (S6 helix) to modify its DAG discrimination capacity by replacing Gly652 with Ala (G652A).^[^ ^39^ ^]^ Human TRPC3 G652A (eYFP‐hTRPC3 G652A) expression plasmid was kindly provided by Dr. Klaus Groschner (University of Graz, Austria). Protein expression of TRPC3 constructs was verified by western blot and static insulin secretion was also studied under these different settings.

### Metabolic Characterization of *Trpc3^−/−^* Mice

Food and water intake were measured by daily weighing of the water bottles and the lab chow (*n* = 50 mice in each group, WT and *Trpc3^−/−^*). The chow (EAN5410340615096, Versele‐Laga, Belgium) composition is detailed in Table S2, Supporting Information, and compared to the chow used previously.^[^ ^54^ ^]^ The standard sucrose preference test was performed in WT and *Trpc3^−/−^* mice (*n* = 25 each) using 1% sucrose and regular water bottles. Insulin sensitivity or tolerance test were performed in 6‐h fasting mice (WT and *Trpc3^−/−^*, *n* = 25 mice each) by injecting i.p. insulin (0.5 U kg^−1^; Humulin R, Eli Lilly, IN, USA). HbA1c plasma concentration was determined by the ELISA technique according to the manufacturer protocol (Mouse glycated hemoglobin A1c Elisa kit, Cusabio, China).

### Western Blot

Tissues and islets were homogenized and lysed in assay lysis buffer (NaCl [150 mm], Tris OH pH 7.5 [50 mm], EDTA [95 mm], triton X‐100 [0.5%]), containing protease and phosphatase inhibitors for protein extraction. The Bradford protein assay (Bio‐Rad, Marnes‐la‐Coquette, France) was used to assess total protein concentrations, and samples were denatured in Laemmli loading buffer (Bio‐Rad, Marnes‐la‐Coquette, France) containing 10% *β*‐mercaptoethanol (Sigma‐Aldrich, St. Louis, MO, USA). Proteins were separated by SDS 10% PAGE then blotted on polyvinylidene fluoride membranes (Bio‐Rad, Marnes‐la‐Coquette, France) already blocked with either 5% or 7% bovine serum albumin. The primary antibodies used were as follows: anti‐mouse and human TRPC3 clone 10H6 (1/1000; MABN748), previously generated by Feng et al.^[^ ^23^ ^]^ and commercialized by Millipore Sigma, MA, USA; anti‐mouse and human TRPC3 (1/1000; ab51560), protein kinase B Pan‐AKT (pan‐Akt) (1/500; ab8805), phospho‐pan‐Akt (p‐Akt T308) (1/500; ab38449), and GAPDH (1/2500; ab9485) (Abcam, Cambridge, UK), and *β*‐actin (1/2000; SAB1305554; Sigma‐Aldrich, St. Louis, MO, USA). The monoclonal anti‐TRPC3 antibody was extensively validated using knockout mice tissues in a previous study.^[^ ^23^ ^]^ The secondary antibodies were goat anti‐rabbit (1/3000) and goat anti‐mouse (1/3000) (Bio‐Rad Laboratories, Marnes‐la‐Coquette, France). Visualization was accomplished using enhanced chemiluminescence (Omega Lum G, Aplegen, Gel Company, SF, USA). Three western blots were performed for each condition.

### Histology and Immunofluorescence

The pancreases, cerebral cortices, hearts, kidneys, livers, and skeletal muscles were fixed in neutral buffered formalin then embedded in paraffin, cut in 4 µm sections, and stained with hematoxylin and eosin (H&E) (Sigma‐Aldrich, St. Louis, MO, USA). Cardiac inflammation, cortical neuronal damage, renal tubular and glomerular changes, and skeletal muscle inflammation were assessed. Representative pictures were taken using a VanGuard High‐Definition Digital Camera (VEE GEE Scientific, Illinois, USA).

Pancreatic sections designated for immunofluorescence were incubated with glycine to lower background. Triton X‐100 was used to achieve permeabilization, and blocking was performed with 10% goat serum and 1% bovine serum albumin. The primary antibodies were: anti‐glucose transporter GLUT2 (ab54460), anti‐mouse and human insulin antibody (ab7842) (Abcam, Cambridge, UK), and anti‐mouse and human TRPC3 clone 10H6 (MABN748) (Millipore Sigma, MA, USA).^[^ ^23^ ^]^ The secondary antibodies were: goat anti‐rabbit IgG H&L Alexa Fluor 594 (ab150084), goat anti‐mouse IgG H&L Alexa Fluor 594 (ab150116), and goat anti‐guinea pig IgG H&L Alexa Fluor 488 (ab150185) (Abcam, Cambridge, UK). Finally, sections were mounted with Fluoroshield mounting medium containing 4′,6‐diamidino‐2‐phenylindole (DAPI) (Abcam, Cambridge, UK), and images were taken using an Axioskop 2 immunofluorescence microscope (Carl Zeiss Microscopy GmbH, Jena, Germany) equipped with a CoolCube 1 CCD camera (MetaSystems, Newton, Massachusetts, USA).

### Calcium Imaging

Isolated islets were cultured in RPMI supplemented with 10% FBS for 24 h. They were then incubated for 1 h at 37 °C with low‐glucose (1.5 mm) Tyrode and additional 45 min at room temperature in the same solution with 4 µm fluo4‐AM (Molecular Probes, Thermo Fisher Scientific, MA, USA). Islets were incubated with the inhibitors (Pyr10, 3 µm; SKF96365 30 µm) and the activator (GSK1702934A 80 nm) from the start till the end of the recordings, much as in the perifusion experiments. A digital fluorescence imaging system was used to capture and interpret images of multiple ROIs (regions of interest) in each islet (InCyt Im2; Intracellular Imaging Inc., Cincinnati, OH, USA). A minimum of 30 recordings were conducted for each condition. Typical calcium recordings were obtained as previously described.^[^ ^30^, ^35^, ^66^ ^]^

### Ex Vivo and In Vitro Glucose Uptake Imaging

Fluorescent glucose, 2‐NBDG (at 200 µm), was used to assess islet and pancreatic glucose uptake as previously described.^[^ ^42^ ^]^ Ex vivo 2‐NBDG pancreatic imaging was performed as previously described.^[^ ^41^ ^]^ 2‐NBDG was injected at 10 mg kg^−1^ i.m. *n* = 5 islets and 5 pancreases for each group.

### Diabetes Model and In Vivo Treatments

Type 2 diabetes was induced in the WT mice by the classic high fat diet coupled to STZ injections. GSK1702934A was administered by osmotic minipumps (Alzet, Durect, CA, USA) placed subcutaneously at a dosage of 0.1 mg kg^−1^ day^−1^ as defined for other related pyrazole compounds.^[^ ^9^, ^14^ ^]^ The protocol lasted 4 weeks. Blood glucose, plasma insulin, HbA1c, IpGTT, and AKT protein activation were assessed as previously described in this paper. Tissues were harvested from the mice to evaluate potential GSK1702934A treatment side effects.

### Diagram Designs

The graphical abstract and Figure S3, Supporting Information, of the manuscript were created with BioRender.com.

### Statistical Analysis

Statistical analysis was conducted using GraphPad Prism 9 and data presented as mean ± SEM. *N* numbers are included in each figure legend. First, when the number of samples per condition was low (*n* = 5–7), Shapiro–Wilk normality test was used to check whether populations followed a Gaussian distribution. When the number of samples per condition was high (*n* ≥ 8), D'Agostino and Pearson normality test was used to check for Gaussian distribution. Then, to test whether populations had comparable variances, Bartlett's test was used when Gaussian distribution was present, whereas Brown–Forsythe test was used when the data were skewed. For comparisons of greater than two groups, and when Gaussian distribution with equal variances were met, ordinary one‐way ANOVA was used for multiple comparisons of the means, followed by post‐hoc Tukey's test to determine which groups accounted for overall ANOVA significance. When Gaussian distribution was present with unequal variances, Brown–Forsythe and Welch ANOVA tests were applied, followed by Dunnett's T3. When data were skewed, Kruskal–Wallis one‐way ANOVA on ranks was used, followed by Dunn's multiple comparisons test. When comparing two‐sample groups, unpaired two‐tailed *t*‐test was used when Gaussian distribution with equal variances were met, whereas Welch's correction was applied with unequal variances. Finally, when two independent parameters were compared, that is, time and treatment, repeated measures two‐way ANOVA w/o the Geisser–Greenhouse correction was performed, followed by Dunnett's or Sidak's multiple comparisons test, with individual variances computed for each comparison. For representation of statistical significance, *, **, ***, and **** indicated *p* values of <0.05, <0.01, <0.001, and <0.0001, respectively. ns: not significant.

### Ethics Approval

The study was approved by the Ethical Committee of the Saint Joseph University. Protocols were designed per the Guiding Principles in the Care and Use of Animals approved by the Council of the American Physiological Society as well as the Guide for the Care and Use of Laboratory Animals published by the US National Institutes of Health (NIH Publication no. 85‐23, revised 1996) and the European Parliament Directive 2010/63 EU. All human subjects gave informed consent. The study on human islets was approved by the Ethical Committee of Saint Joseph University and the reported investigations were carried out in accordance with the principles of the Declaration of Helsinki as revised in 2008. The subjects’ and islets’ characteristics are listed in Table S1, Supporting Information.

## Conflict of Interest

The authors declare no conflict of interest.

## Supporting information

Supporting Information

Supporting Information

Supporting Information

Supporting Information

## Data Availability

The data that support the findings of this study are available on request from the corresponding author. The data are not publicly available due to privacy or ethical restrictions.
