# Supplementary material for: TRPC3 Regulates Islet Beta‐Cell Insulin Secretion
Source: Adv Sci (Weinh). 2023 Jan 15;10(6):2204846. doi: 10.1002/advs.202204846 (PMC9951314; doi:10.1002/advs.202204846)
Supplement: Supplementary file 1 — Supporting Information [file ADVS-10-2204846-s002.pdf]

ADVANCED SCIENCE
Open Access

Supporting Information

for *Adv. Sci.*, DOI 10.1002/advs.202204846

TRPC3 Regulates Islet Beta-Cell Insulin Secretion

Gaëlle Rached, Youakim Saliba, Dina Maddah, Joelle Hajal, Viviane Smayra, Jules-Joel Bakhos, Klaus Groschner, Lutz Birnbaumer and Nassim Fares**

Supplementary Information

Human islets

All human subjects gave informed consent. The study was approved by the Ethical Committee of Saint Joseph University and the reported investigations have been carried out in accordance with the principles of the Declaration of Helsinki as revised in 2008. The subjects' and islets' characteristics are listed in Supplemental Table 1.

Animals

The study was approved by the Ethical Committee of the Saint Joseph University. Protocols were designed per the Guiding Principles in the Care and Use of Animals approved by the Council of the American Physiological Society as well as the Guide for the Care and Use of Laboratory Animals published by the US National Institutes of Health (NIH Publication no. 85-23, revised 1996) and the European Parliament Directive 2010/63 EU. The study was carried out on male TRPC3 knockout (*Trpc3*$^{-/-}$) mice aged 2 and 14 months with their age-matched littermate wild-type (WT) controls. Mice were developed at the Comparative Medicine Branch of the NIEHS on a 129SvEv/C57BL/6J mixed background by deleting exon 7 of the *Trpc3* gene in a three-step process [1] and were obtained from Pr Nancy Rusch's laboratory stock at the University of Arkansas for Medical Sciences and bred in-house. Animals were housed at a stable temperature (25°C) and humidity (50 ± 5%) and were exposed to a 12:12h light-dark cycle. They were fed standard rodent chow and had free access to tap water.

As shown in the Supplemental Figure 1 flowchart, TRPC3 expression experiments on human and mouse beta cells were matched with *in vivo* intraperitoneal (i.p.) glucose and arginine tolerance tests on WT mice w/o a TRPC3-specific inhibitor and *Trpc3*$^{-/-}$ mice. TRPC3-dependent GSIS was subsequently assessed using various settings in human and mouse isolated islets.

Additionally, TRPC3-dependent *in vivo* insulin secretion was studied in mice. Following that, *Trpc3*$^{-/-}$ mice were metabolically characterized and *ex vivo* and *in vitro* glucose uptake were evaluated. Moreover, calcium imaging was performed on isolated mouse islets to study the role of TRPC3 in glucose-stimulated calcium (GSCa) oscillations. Finally, the therapeutic potential of TRPC3 modulation by a specific small-molecule activator was investigated in a diabetic mouse model.

Intraperitoneal glucose tolerance tests (IpGTT)

The IpGTT was conducted instead of the OGTT because of its ease of use, while also being less stressful for animals than the intragastric gavage technique during OGTT. Add to that, it circumvents the incretin response that might obscure GSIS. The IpGTT was first carried out on three groups of 25 mice per group (three independent experiments): WT, WT with Pyr10 (specific TRPC3 inhibitor) [2], and *Trpc3*$^{-/-}$ mice. Pyr10 (N-[4-[3,5-Bis(trifluoromethyl)-1H-pyrazol-1-yl]phenyl]-4-methyl-benzenesulfonamide) (Sigma-Aldrich, St. Louis, MO, USA) was acutely administered (i.p.) 10 minutes prior to the IpGTT, at a dose of 8 $\mu g.kg^{-1}$ by analogy to previously described daily doses [3,4]. IpGTT was performed after a six-hour fast, allowing for a proper assessment of glucose resistance without subjecting the animals to unnecessary starvation [5]. Mice were given i.p. injections of 2 $g.kg^{-1}$ glucose after a six-hour fast that began at 7 a.m. Conscious mice were then put into rodent plexiglass restrainers (IITC Life Science Inc., CA, USA) and blood glucose levels were then measured from the tail with a glucometer (Accu-Chek, Roche Diabetes Care, IN, USA) at T = 0 minutes just before glucose injection, as well as 15 minutes, 30 minutes, 60 minutes, and 120 minutes later. Tails were first wiped with 70% alcohol, and the second drop of blood was used for the measurements. For TRPC3 activation testing, two additional mouse groups were used: WT (n=11) and WT acutely administered (i.p.) with a

specific TRPC3 small-molecule activator GSK1702934A [6] (kindly provided by Pr. Klaus Groschner, Gottfried-Schatz-Research-Centre-Biophysics, Medical University of Graz, Austria) at a dose of 35 µg.kg^{-1} (n=11). Doses ranging from 35 to 500 µg.kg^{-1} were tested, and the lower dose was chosen. GSK1702934A was acutely administered (i.p.) 10 minutes prior to the IpGTT. Since Pyr10 and GSK1702934A are dissolved in DMSO, WT were injected with equal DMSO volumes as negative controls.

As for IpGTT during TRPC6 inhibition, SAR7334 was used at a dose of 5 mg.kg^{-1} ensuring TRPC6 maximal inhibition without interfering with the activities of TRPC3 and TRPC7 [7]. SAR7334 was administered i.p. one hour before the start of the experiment.

Plasma insulin during IpGTT and arginine challenge

Blood samples were collected from the restrained mice during the IpGTT in EDTA mini-tubes, and hemolyzed samples were discarded to avoid insulin degradation by insulin-degrading enzymes [8]. Similarly, insulin secretion was assessed following an i.p. injection of arginine (1 g.kg^{-1}) in another set of mice: WT (n = 25), WT previously administered with Pyr10 (n=25), and *Trpc3*$^{-/-}$ mice (n = 25) (three independent experiments). Arginine stimulates insulin secretion through stimulation of membrane depolarization [9,10]. Plasma insulin was then measured according to Crystal Chem's Ultra-Sensitive Mouse Insulin ELISA Kit (IL, USA) with a broad dynamic range (0.4 - 64 ng.mL^{-1}) and high sensitivity requiring only a 5 µL sample per test. Measurements were performed at 0, 15, 30, 60, and 120 minutes after glucose or arginine injection.

Islet and beta-cell isolation and culture

Animals were anesthetized by a mixture of ketamine (75 mg.kg^{-1}; Interchemie, Waalre, Holland) and xylazine (10 mg.kg^{-1}; RotexMedica, Trittau, Germany). When the animals were totally non-

responsive to toe pinching, an abdominal incision was made aseptically, and the pancreases were harvested while preventing the collection of fat tissue. Pancreases were then rinsed with cold modified Tyrode free-calcium solution containing (in $mmol.L^{-1}$): 140 NaCl, 5.7 KCl, 1.7 $MgCl_2$, 4.4 $NaHCO_3$, 1.5 KH_2PO_4, 10 HEPES, 10 creatine monohydrate, 20 taurine, 11.7 d-glucose, pH 7.2 adjusted with NaOH. They were carefully cut into tiny (2 mm) parts in modified Tyrode and washed several times in this solution to remove any blood or fat tissue contamination. Following that, tissues were digested for 60 minutes in an oxygenated enzymatic bath (100 rpm shaker at 37°C). The bath contained 1 $mg.mL^{-1}$ collagenase A (Roche Diagnostics, Germany) that possesses a low collagenase activity (>0.15 $U.mg^{-1}$) with 1 $mg.mL^{-1}$ bovine serum albumin (Sigma Aldrich, St. Louis, Missouri, USA). Digestion was stopped by the addition of an equal volume of ice-cold modified Tyrode and the tissue solution was vigorously hand-shaken to mechanically dissociate the digested islets. After that, the solution was put on ice for 3 minutes to sediment the islets, and the process was repeated three times. To further purify the islets from contaminating exocrine tissue, a Percoll solution (Sigma Aldrich, St. Louis, Missouri, USA) with a density of 1.045 $g.mL^{-1}$ was used. To begin, a stock isotonic Percoll (SIP) solution was prepared by mixing 9 parts of Percoll with 1 part of 1.5 $mol.L^{-1}$ NaCl (10X concentrated), then this solution was diluted to 1.045 $g.mL^{-1}$ by adding 0.15 $mol.L^{-1}$ NaCl using the following formula: $V_y = V_i \times (\rho_i - \rho) / (\rho - \rho_y)$ where V_y is the volume of diluting 0.15 $mol.L^{-1}$ NaCl, V_i is the volume of SIP, ρ_i is the density of SIP, ρ_y is the density of 0.15 $mol.L^{-1}$ NaCl and ρ is the density of final diluted Percoll solution. The digested pancreatic tissue was carefully placed on top of the 1.045 $g.mL^{-1}$ Percoll solution and allowed to sediment for 5 minutes. Islets were collected from the bottom of the tube and this step was repeated 3 times. Finally, the islets were cultured in RPMI 1640 (Lonza, Basel, Switzerland) supplemented with 2 $mmol.L^{-1}$ L-glutamine,

10% FBS and 1% penicillin/streptomycin for 24 hours before the treatments. When isolated beta cells were needed, islets were further dispersed with 0.25% trypsin for 10 min at 37 °C before incubating the cells in RPMI 1640.

Human pancreatic samples were collected from the normal tissue of patients (n=4) undergoing the Whipple Procedure for pancreatic adenocarcinoma resection. The tissues were divided in two, with one half being used to isolate islets and the other half being stored in neutral buffered formalin for future immunofluorescence studies. The same method was used to separate human islets as it was for mice, with an additional hand-picking step.

Islet treatment protocols

Islets were isolated and cultured from human and mouse pancreatic tissues as mentioned above. Islets were then switched from RPMI to low-glucose (1.5 mM) Tyrode and incubated in this solution for one hour to establish a baseline insulin secretion. Afterward, the WT islets were treated with Pyr10 (3 µM), a non-specific Ca^{2+} entry blocker SKF96365 (30 µM), or GSK1702934A (80 nM) for ten minutes prior to starting the perifusion experiments and till the end. GSK1702934A concentration was chosen by *in vivo*/*in vitro* conversion according to the 35 $\mu g.kg^{-1}$ dose used in the IpGTT. Five islets were studied for each condition: WT control (DMSO), WT Pyr10, WT SKF96365, GSK1702934A, and *Trpc3*$^{-/-}$. Islets were mounted in a Siskiyou perfusion chamber (Automate Scientific, CA, USA) then perfused with the different solutions using a ValveLink8.2 Perfusion Controller (Automate Scientific, CA, USA). Solutions were then recovered using an Instech P720 peristaltic pump (Instech, PA, USA) every 2.5 minutes. Islet insulin secretion was measured at baseline glucose concentration (1.5 mM) for a duration of five minutes followed by either 8 mM glucose or a higher concentration of 16.7 mM for 40 minutes. A final KCl (30 mM) infusion was used to completely depolarize the islets and

check for total insulin content. For arginine (20 mM) and KCl (30 mM) + diazoxide (100 µM) testing, static insulin secretion was studied for 20 minutes. The islet insulin secretion was measured using Crystal Chem's Ultra-Sensitive Mouse Insulin ELISA Kit (IL, USA) using only a 5 µL sample, and the Human Insulin ELISA kit (ab100578; Abcam, Cambridge, USA).

Metabolic characterization of Trpc3$^{-/-}$ mice

Food and water intake were measured by weighing the water bottles as well as the lab chow daily prior and after consumption (n=50 mice in each group, WT and *Trpc3*$^{-/-}$). The chow (EAN5410340615096, Versele-Laga, Belgium) composition is detailed in Supplemental Table 2 and compared to the chow used previously [11]. Where applicable, vitamin E (α-tocopherol) was administered to mice in the drinking bottle at a dose of 5 I.U. per day. In the sucrose preference test, WT and *Trpc3*$^{-/-}$ mice (n=25 each) were given access to two standard-sized bottles, one containing 1% sucrose and the other containing regular water, for the first four days. After that, bottles were given for additional 48 hours, and sucrose intake was then recorded [12]. Mice had unrestricted access to lab chow all along. Insulin sensitivity or tolerance test were performed in 6-hour fasting mice (WT and *Trpc3*$^{-/-}$, n=25 mice each) by injecting i.p. insulin (0.5 $U.kg^{-1}$; Humulin R, Eli Lilly, IN, USA) then testing blood glucose as previously mentioned. Finally, HbA1c plasma concentration was determined by the ELISA technique according to the manufacturer protocol (Mouse glycated hemoglobin A1c Elisa kit, Cusabio, China).

Primary cultures of mediobasal hypothalamic (MBH) neurons

MBH neurons were prepared from the brains of C57BL/6J mice with slight modifications of the previously described procedures [13–16]. C57BL/6J mice were anesthetized with the ketamine/xylazine mixture. Brains were extracted and put in ice-cold oxygenated (95% O_2 / 5% CO_2) perfusion buffer (in $mmol.L^{-1}$: 2.5 KCl, 1.25 NaH_2PO_4, 28 $NaHCO_3$, 7 $MgCl_2$, 0.5 $CaCl_2$, 7

glucose, 1 ascorbate, and 3 pyruvate). Using a Vibratome (Leica Biosystems, Wetzlar, Germany), coronal hypothalamic sections (500 μm) were cut. Hypothalamic sections were then maintained at 34°C in oxygenated artificial cerebrospinal fluid containing 2.5 mM glucose (in mM: 127 NaCl, 1.9 KCl, 1.2 KH_2PO_4, 26 $NaHCO_3$, 1.3 $MgCl_2$, and 2.4 $CaCl_2$, pH 7.4, with osmolarity adjusted to ~300 $mosmol.kg^{-1}$ H_2O with sucrose) for 30 min. MBH was then dissected from coronal slices by using a magnifying loupe and cutting out an equilateral triangle. MBH sections were then digested in artificial cerebrospinal fluid with papain (Sigma-Aldrich, St. Louis, MO, USA; final concentration 20 $U.mL^{-1}$) for 30 min in a 37°C rotating platform water bath at 100 rpm. The tissue was cleansed with the artificial cerebrospinal fluid and then gently triturated. After trituration, the cell suspension was centrifuged and the pellet resuspended in Neurobasal Plus growth medium (ThermoFisher Scientific, MA, USA) containing 10% FBS, 1% penicillin/streptomycin, 10 $\mu g.mL^{-1}$ gentamycin, and 1% B-27 (ThermoFisher Scientific, MA, USA). Cells were plated on poly-D-lysine (50 $\mu g.mL^{-1}$)-coated glass coverslips in the complete Neurobasal Plus growth medium overnight before calcium imaging experiments. Changes in intracellular calcium were quantitated by calculating the area under the curve (AUC) of each glucose response.

Histology and immunofluorescence

The pancreases, cerebral cortices, hearts, kidneys, livers, and skeletal muscles were harvested from mice as previously mentioned. Neutral buffered formalin was used with a pH of 7.0 stabilized by the addition of sodium dihydrogen phosphate monohydrate ($NaH_2PO_4.H_2O$) and disodium hydrogen phosphate anhydrous (Na_2HPO_4). For histopathological examination, fixed pancreatic tissue was embedded in paraffin, cut in 4 μm sections, and stained with hematoxylin and eosin (H&E) (Sigma-Aldrich, St. Louis, MO, USA). Other section series was also obtained

for later immunofluorescence experiments. After H&E staining, sections were rinsed in distilled water, dehydrated in ethanol/water baths with decreasing water content, and finally rinsed in xylene before being mounted with a permanent mounting medium. Two different pathologists performed histological studies. Cardiac inflammation was assessed by evaluating the numbers of infiltrating leukocytes in the cardiac tissue. Cortical neuronal damage, such as shrinkage and scalloping (pyknosis) of dying neurons, was evaluated as previously described [17]. Furthermore, renal tubular injury (i.e. necrosis), glomerular diameter, and skeletal muscle inflammation (leukocyte infiltration) were assessed. Representative pictures were at last taken using a VanGuard High-Definition Digital Camera (VEE GEE Scientific, Illinois, USA). Islet sizes were calculated using ImageJ software. Two sections and two view fields were analyzed in each condition in human and animal tissues.

Pancreatic sections designated for immunofluorescence were incubated with 0.3 M glycine for 20 minutes at room temperature, which binds free aldehyde groups that would otherwise bind the primary and secondary antibodies, leading to high background. Triton X-100 was used for 20 minutes at room temperature to achieve permeabilization. Blocking was performed with 10% goat serum and 1% bovine serum albumin diluted in phosphate buffer saline for one hour at 37°C. The primary antibodies were incubated overnight in blocking buffer at 4°C. Antibodies were: anti-glucose transporter GLUT2 (ab54460), anti-mouse and human insulin antibody (ab7842), (Abcam, Cambridge, UK), and anti-mouse and human TRPC3 clone 10H6 (MABN748) previously generated by Feng et al. [18] and commercialized by Millipore Sigma, MA, USA. The following day, sections were washed with phosphate buffer saline then incubated with the secondary antibodies for 30 minutes at 37°C; secondary antibodies were: goat anti-rabbit IgG H&L Alexa Fluor 594 (ab150084), goat anti-mouse IgG H&L Alexa Fluor 594

(ab150116), and goat anti-guinea pig IgG H&L Alexa Fluor 488 (ab150185) (Abcam, Cambridge, UK). To reduce background noise and lower the risk of cross-reactivity between secondary antibodies and endogenous proteins and immunoglobulins, the secondary antibodies were pre-adsorbed by passing them through a column matrix containing immobilized serum proteins from the same species the tissue samples originated from i.e., mouse. Finally, sections were mounted with Fluoroshield Mounting Medium containing 4′,6-diamidino-2-phenylindole (DAPI) (Abcam, Cambridge, UK), and images were taken using an Axioskop 2 immunofluorescence microscope (Carl Zeiss Microscopy GmbH, Jena, Germany) equipped with a CoolCube 1 CCD camera (MetaSystems, Newton, Massachusetts, USA). Image processing was carried out using ImageJ. Two sections were analyzed in each condition.

Ex vivo and in vitro glucose uptake imaging

Fluorescent glucose, 2-[N-(7-nitrobenz-2-oxa-1,3-diazol-4-yl) amino]-2-deoxy-D-glucose (2-NBDG), was used to assess islet and pancreatic glucose uptake. Isolated and cultured islets were washed with glucose-free Tyrode, and 2-NBDG was applied to the cells at a final concentration of 200 μM for 5 min at 37 °C [19]. Islets were then washed with glucose-free Tyrode to eliminate background fluorescence, and an image was acquired. The entire procedure was repeated four times for a total exposure of 20 minutes. 2-NBDG was excited at 465-495 nm and emission collected at 515–555 nm. Fluorescence intensity at each point was determined using ImageJ; after background subtraction, fluorescence intensity was calculated as the difference in the average fluorescence of cells before and after each application of 2-NBDG.

Ex vivo 2-NBDG pancreatic imaging was performed as previously described [20]. Briefly, fasting mice were sedated then the abdominal incision was performed to expose the pancreas. Pancreatic tissue was extended and incubated in a petri dish with a Tyrode solution. 2-NBDG was also

injected 10 $mg.kg^{-1}$ i.m. in 100 µl of 0.9% NaCl. Fluorescence increase over time in ROIs was measured. Background figures were acquired just before 2-NBDG injections. n= 5 islets and 5 pancreases for each group.

Diabetes model and in vivo treatments

Type 2 diabetes was induced by the classic high fat diet coupled to streptozotocin (STZ) injections [21,22]. Mice were given a high-fat diet (60% kcal as fat from lard) for four weeks before receiving three i.p. streptozotocin injections (40 $mg.kg^{-1}$ each) on three consecutive days. STZ was diluted with a citrate buffer at pH = 4.5. To ensure adequate diabetes induction, blood glucose was tested 72 hours after the third STZ injection. The selective TRPC3 activator GSK1702934A was administered by osmotic minipumps (Alzet, Durect, CA, USA) placed subcutaneously on the back of the mice slightly posterior to the scapulae at a dosage of 0.1 $mg.kg^{-1}.day^{-1}$ as defined for other related pyrazole compounds [3,4]. The protocol lasted four weeks. Blood glucose, plasma insulin, HbA1c, IpGTT, and AKT protein activation were assessed as previously described in this paper. Tissues were harvested from the mice to evaluate potential GSK1702934A treatment side effects.

Supplemental Fig. 1. Descriptive diagram depicting the experiments carried out in this study. TRPC3 expression in human and mouse beta cells was assessed, followed by intraperitoneal glucose tolerance tests (IpGTT) in three groups of mice: WT, WT Pyr10, and *Trpc3*$^{-/-}$. Insulin secretion *in vitro* was assessed using glucose-stimulated insulin secretion (GSIS) in human and mouse isolated islets in various settings. The metabolic characterization of *Trpc3*$^{-/-}$ mice was then carried out. *Ex vivo* and *in vitro* glucose uptake, as well as *in vivo* insulin secretion, were also assessed. After that, calcium imaging on isolated islets was performed. Finally, the impact of pharmacologic TRPC3 activation in diabetes was investigated. The flowchart design was created in BioRender.com

Supplemental Fig. 2. TRPC3 and ROS interplay in glucose-excited (GE) neurons of the mediobasal hypothalamus (MBH) regulate food intake in mice. a-d: Representative calcium and ROS imaging traces of MBH GE neuron response to high glucose from WT mice w/o α-tocopherol treatment. **e-h**: Representative calcium and ROS imaging traces of MBH GE neuron response to high glucose from *Trpc3*$^{-/-}$ mice w/o α-tocopherol treatment. **i, j**: Quantifications of the calcium and ROS responses to glucose stimulation in MBH neurons. n=15 neuron cells from three independent experiments for each condition. G2.5: glucose 2.5 mM, and G10: glucose 10 mM. α-tocopherol (10 μM). *** $p < 0.001$, and **** $p < 0.0001$ *vs* WT. **k**: Food intake of WT and *Trpc3*$^{-/-}$ mice w/o α-tocopherol treatment. α-tocopherol was administered *per os*. n=6 mice in each condition. *** $p < 0.001$, and **** $p < 0.0001$ *vs* WT before and after treatment with α-

tocopherol, and *vs Trpc3*$^{-/-}$. One-Way ANOVA were used followed by Tukey's tests in (i, j). Repeated Measures Two-Way ANOVA were used followed by Sidak's tests in (k).

Supplemental Fig. 3. Glucose-stimulated insulin secretion is regulated by TRPC3. TRPC3's role in glucose-stimulated insulin release in pancreatic beta cells is depicted in this diagram (created with BioRender.com). GLUT2: Glucose transporter 2; K_{ATP}: ATP-dependent potassium channel; LTCC: L-type calcium channel; DAG: Diacylglycerol; TRPC3: Transient receptor potential canonical 3.

**Islet preparation**	**1**	**2**	**3**	**4**
Donor age (years)	76	82	54	24
Donor sex (M/F)	F	F	M	F
Donor BMI (kg/m^2)	26.22	24.17	24.54	21.25
Donor HbA_{1c} or other measure of blood glucose control	4.8	6.5	4.9	4.6
Origin/source of islets	Pancreatic biopsy	Pancreatic biopsy	Pancreatic biopsy	Pancreatic biopsy
Islet isolation centre	Hôtel-Dieu de France Hospital , Beirut, Lebanon	Hôtel-Dieu de France Hospital , Beirut, Lebanon	Hôtel-Dieu de France Hospital , Beirut, Lebanon	Hôtel-Dieu de France Hospital , Beirut, Lebanon
Donor history of diabetes? Please select yes/no from drop down list	No	Yes	No	No
Diabetes duration (years)		2		
Glucose-lowering therapy at time of death		Metformin 850 mg		
Donor cause of death	N.A.	N.A.	N.A.	N.A.
Warm ischaemia time (h)	N.A.	N.A.	N.A.	N.A.
Cold ischaemia time (h)	N.A.	N.A.	N.A.	N.A.
Estimated purity (%)	>90%	>90%	>90%	>90%
Estimated viability (%)	>90%	>90%	>90%	>90%
Total culture time (h)	30 minutes from the time of biopsy to the receiving laboratory; 24 hours at the	30 minutes from the time of biopsy to the receiving laboratory; 24 hours at the	30 minutes from the time of biopsy to the receiving laboratory; 24 hours at the	30 minutes from the time of biopsy to the receiving laboratory; 24 hours at the

	receiving laboratory (in RPMI + FBS)	receiving laboratory (in RPMI + FBS)	receiving laboratory (in RPMI + FBS)	receiving laboratory (in RPMI + FBS)
Handpicked to purity? Please select yes/no from drop down list	Yes	Yes	Yes	Yes

Supplemental Table 1. Human subjects' and islets' characteristics.

Supplemental Table 2. Rodent chow composition.

	EAN5410340615096 (Versele Laga)	Safe A04 (Safe Diets)
%		
Protein	20	16.1
Fat	4	3.1
Fiber	4	3.9
Ash	7	4.6
Calcium	1.1	0.73
Phosphorus	0.8	0.55
$.kg^{-1}$		
A	18000 IU	7500 IU
D3	2150 IU	1000 IU
E	140 mg (≈209 IU)	30 IU
Iron	180 mg	270 mg
Iodine	3.5 mg	-
Copper	18 mg	16 mg
Manganese	135 mg	70 mg

Zinc	125 mg	55 mg
Selenium	0.35 mg	-

Supplemental References

[1] J. Hartmann, E. Dragicevic, H. Adelsberger, H. A. Henning, M. Sumser, J. Abramowitz, R. Blum, A. Dietrich, M. Freichel, V. Flockerzi, L. Birnbaumer, A. Konnerth, *Neuron* **2008**, *59*, 392.

[2] H. Schleifer, B. Doleschal, M. Lichtenegger, R. Oppenrieder, I. Derler, I. Frischauf, T. Glasnov, C. Kappe, C. Romanin, K. Groschner, *British Journal of Pharmacology* **2012**, *167*, 1712.

[3] Y. Saliba, V. Jebara, J. Hajal, R. Maroun, S. Chacar, V. Smayra, J. Abramowitz, L. Birnbaumer, N. Farès, *Antioxidants & Redox Signaling* **2019**, *30*, 1851.

[4] S. Kiyonaka, K. Kato, M. Nishida, K. Mio, T. Numaga, Y. Sawaguchi, T. Yoshida, M. Wakamori, E. Mori, T. Numata, M. Ishii, H. Takemoto, A. Ojida, K. Watanabe, A. Uemura, H. Kurose, T. Morii, T. Kobayashi, Y. Sato, C. Sato, I. Hamachi, Y. Mori, *Proceedings of the National Academy of Sciences* **2009**, *106*, 5400.

[5] S. Andrikopoulos, A. R. Blair, N. Deluca, B. C. Fam, J. Proietto, *American Journal of Physiology-Endocrinology and Metabolism* **2008**, *295*, E1323.

[6] B. Doleschal, U. Primessnig, G. Wolkart, S. Wolf, M. Schernthaner, M. Lichtenegger, T. N. Glasnov, C. O. Kappe, B. Mayer, G. Antoons, F. Heinzel, M. Poteser, K. Groschner, *Cardiovascular Research* **2015**, *106*, 163.

[7] T. Maier, M. Follmann, G. Hessler, H.-W. Kleemann, S. Hachtel, B. Fuchs, N. Weissmann, W. Linz, T. Schmidt, M. Löhn, K. Schroeter, L. Wang, H. Rütten, C. Strübing, *Br J Pharmacol* **2015**, *172*, 3650.

[8] D. Chevenne, A. Letailleur, F. Trivin, D. Porquet, *Clin Chem* **1998**, *44*, 354.

[9] P. Thams, K. Capito, *European Journal of Endocrinology* **1999**, 87.

[10] R. P. Robertson, R. H. Raymond, D. S. Lee, R. A. Calle, A. Ghosh, P. J. Savage, S. S. Shankar, M. T. Vassileva, G. C. Weir, D. A. Fryburg, Beta Cell Project Team of the Foundation for the NIH Biomarkers Consortium, *Am J Physiol Endocrinol Metab* **2014**, *307*, E720.

[11] C. Chrétien, C. Fenech, F. Liénard, S. Grall, C. Chevalier, S. Chaudy, X. Brenachot, R. Berges, K. Louche, R. Stark, E. Nédélec, A. Laderrière, Z. B. Andrews, A. Benani, V. Flockerzi, J. Gascuel, J. Hartmann, C. Moro, L. Birnbaumer, C. Leloup, L. Pénicaud, X. Fioramonti, *Diabetes* **2017**, *66*, 314.

[12] M.-Y. Liu, C.-Y. Yin, L.-J. Zhu, X.-H. Zhu, C. Xu, C.-X. Luo, H. Chen, D.-Y. Zhu, Q.-G. Zhou, *Nat Protoc* **2018**, *13*, 1686.

[13] A. A. Dunn-Meynell, V. H. Routh, L. Kang, L. Gaspers, B. E. Levin, *Diabetes* **2002**, *51*, 2056.

[14] R. P. Vazirani, X. Fioramonti, V. H. Routh, *J Vis Exp* **2013**, DOI 10.3791/50861.
[15] L. Sousa-Ferreira, A. R. Álvaro, C. Aveleira, M. Santana, I. Brandão, S. Kügler, L. P. de Almeida, C. Cavadas, *PLoS One* **2011**, *6*, e19745.
[16] N. Heeley, P. Kirwan, T. Darwish, M. Arnaud, M. L. Evans, F. T. Merkle, F. Reimann, F. M. Gribble, C. Blouet, *Mol Metab* **2018**, *10*, 14.
[17] P. Rahaman, M. R. Del Bigio, *Acad Forensic Pathol* **2018**, *8*, 539.
[18] S. Feng, H. Li, Y. Tai, J. Huang, Y. Su, J. Abramowitz, M. X. Zhu, L. Birnbaumer, Y. Wang, *Proc Natl Acad Sci U S A* **2013**, *110*, 11011.
[19] K. Yamada, M. Saito, H. Matsuoka, N. Inagaki, *Nat Protoc* **2007**, *2*, 753.
[20] A. Michau, D. J. Hodson, P. Fontanaud, A. Guillou, G. Espinosa-Carrasco, F. Molino, C. J. Peters, I. C. Robinson, P. Le Tissier, P. Mollard, M. Schaeffer, *Diabetes* **2016**, *65*, 463.
[21] E. R. Gilbert, Z. Fu, D. Liu, *Exp Diabetes Res* **2011**, *2011*, 416254.
[22] M. Zhang, X.-Y. Lv, J. Li, Z.-G. Xu, L. Chen, *Exp Diabetes Res* **2008**, *2008*, 704045.
