# Supplementary figures and images for: TRPC3 Regulates Islet Beta‐Cell Insulin Secretion
Source: Adv Sci (Weinh). 2023 Jan 15;10(6):2204846. doi: 10.1002/advs.202204846 (PMC9951314; doi:10.1002/advs.202204846)

## Slide 1
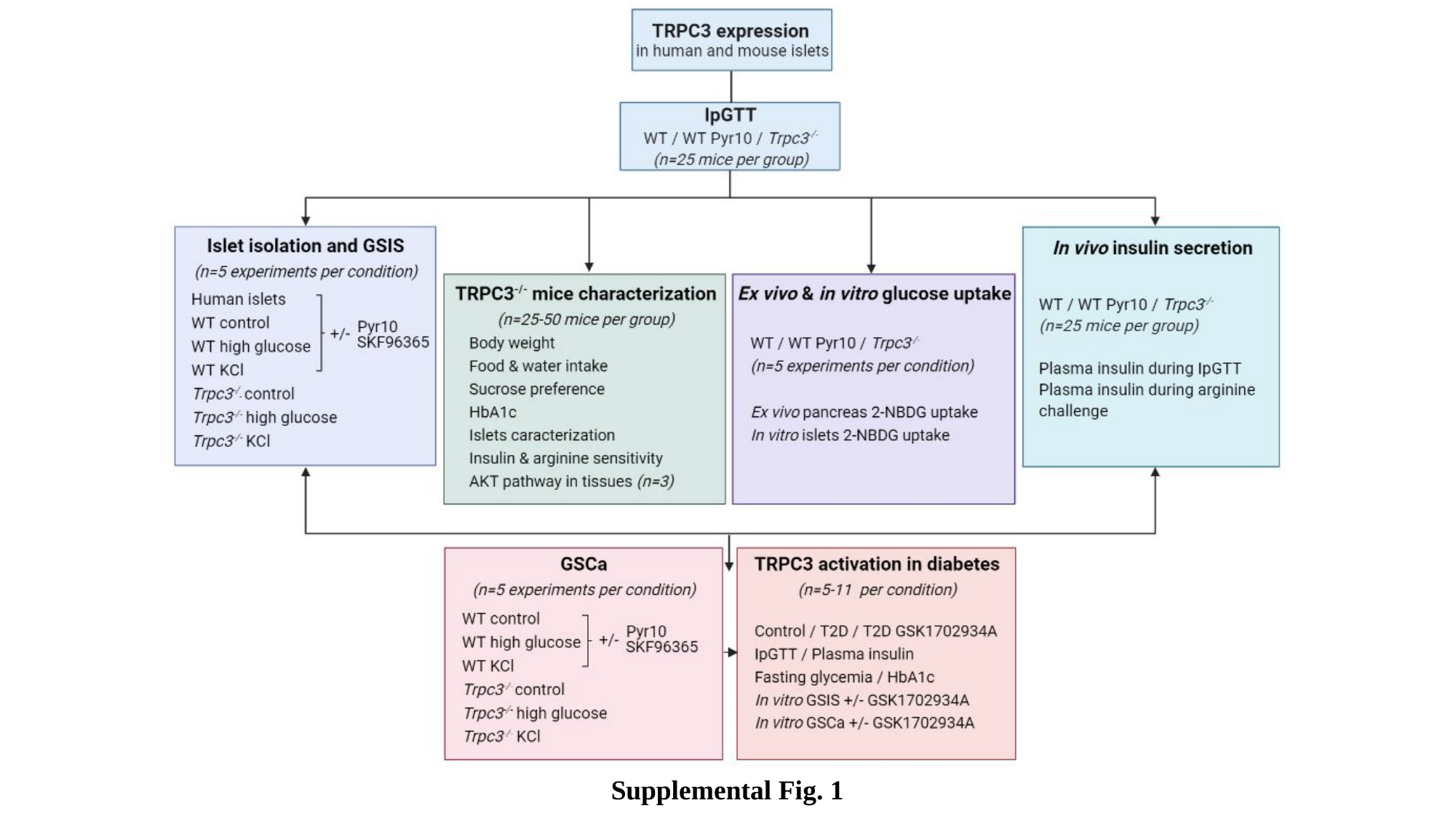

Supplemental Fig. 1

Supplement: Supplementary file 2 — Supporting Information [file ADVS-10-2204846-s004.pptx]
