# Supplementary material for: TRPC3 Regulates Islet Beta‐Cell Insulin Secretion
Source: Adv Sci (Weinh). 2023 Jan 15;10(6):2204846. doi: 10.1002/advs.202204846 (PMC9951314; doi:10.1002/advs.202204846)
Supplement: Supplementary file 3 — Supporting Information [file ADVS-10-2204846-s003.pptx]

## Slide 1
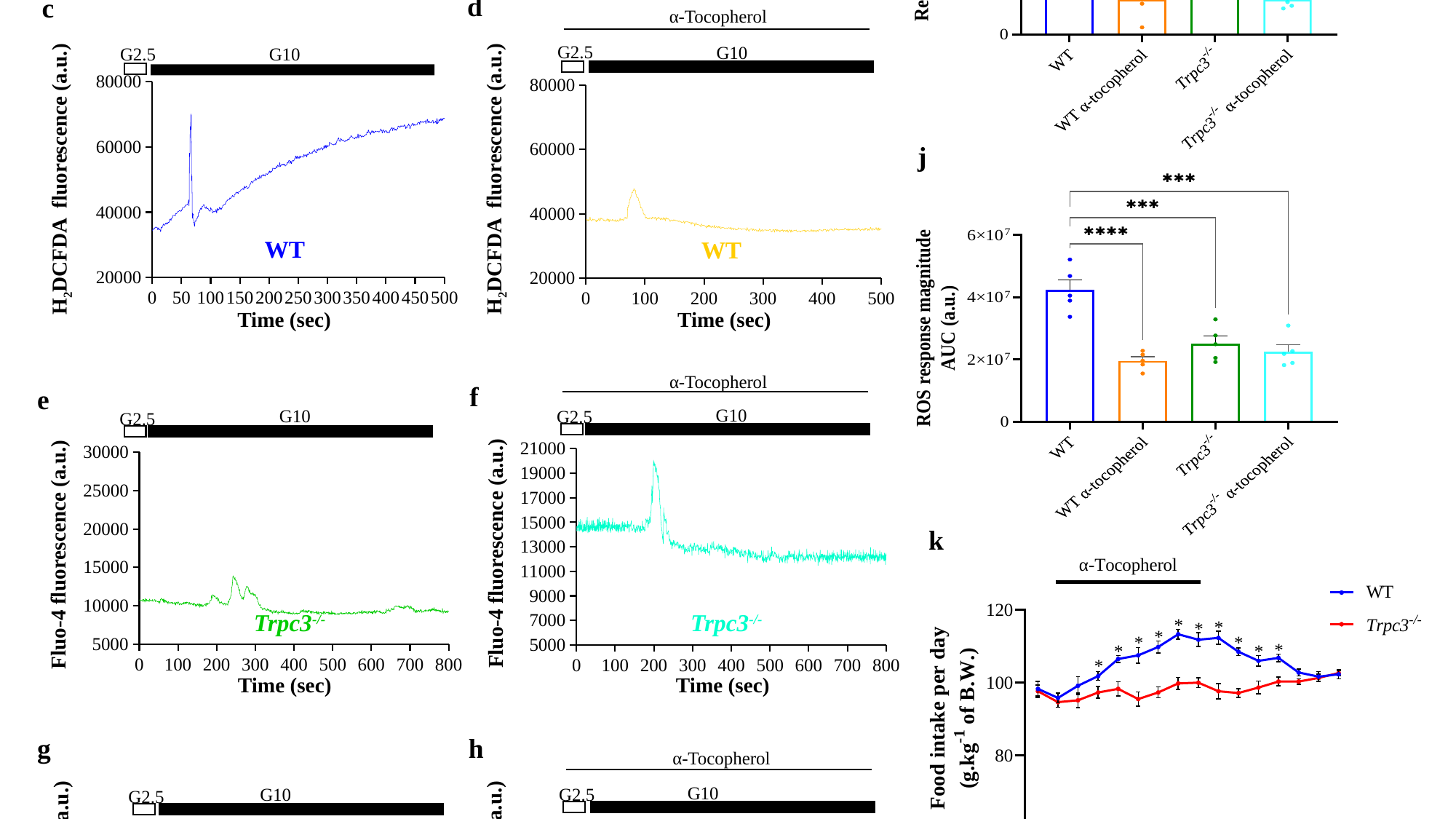

b
α-Tocopherol
a
G2.5
G10
G10
G2.5
### Chart
| Category | |
|---|---|
### Chart
| Category | |
|---|---|
i
Fluo-4 fluorescence (a.u.)
Fluo-4 fluorescence (a.u.)
WT
WT
Time (sec)
Time (sec)
d
c
α-Tocopherol
G2.5
G10
G10
G2.5
### Chart
| Category | |
|---|---|
### Chart
| Category | |
|---|---|j
H2DCFDA fluorescence (a.u.)
H2DCFDA fluorescence (a.u.)
WT
WT
Time (sec)
Time (sec)
α-Tocopherol
f
e
G10
G10
G2.5
G2.5
### Chart
| Category | Object[02] |
|---|---|
### Chart
| Category | Object[09] |
|---|---|k
Fluo-4 fluorescence (a.u.)
Fluo-4 fluorescence (a.u.)
Trpc3-/-
Trpc3-/-
Time (sec)
Time (sec)
g
h
α-Tocopherol
G10
G10
G2.5
G2.5
### Chart
| Category | |
|---|---|
### Chart
| Category | |
|---|---|H2DCFDA fluorescence (a.u.)
H2DCFDA fluorescence (a.u.)
Trpc3-/-
Trpc3-/-
Time (sec)
Time (sec)
Supplemental Fig. 2
