# Supplementary material for: TRPC3 Regulates Islet Beta‐Cell Insulin Secretion
Source: Adv Sci (Weinh). 2023 Jan 15;10(6):2204846. doi: 10.1002/advs.202204846 (PMC9951314; doi:10.1002/advs.202204846)
Supplement: Supplementary file 4 — Supporting Information [file ADVS-10-2204846-s001.pptx]

## Slide 1
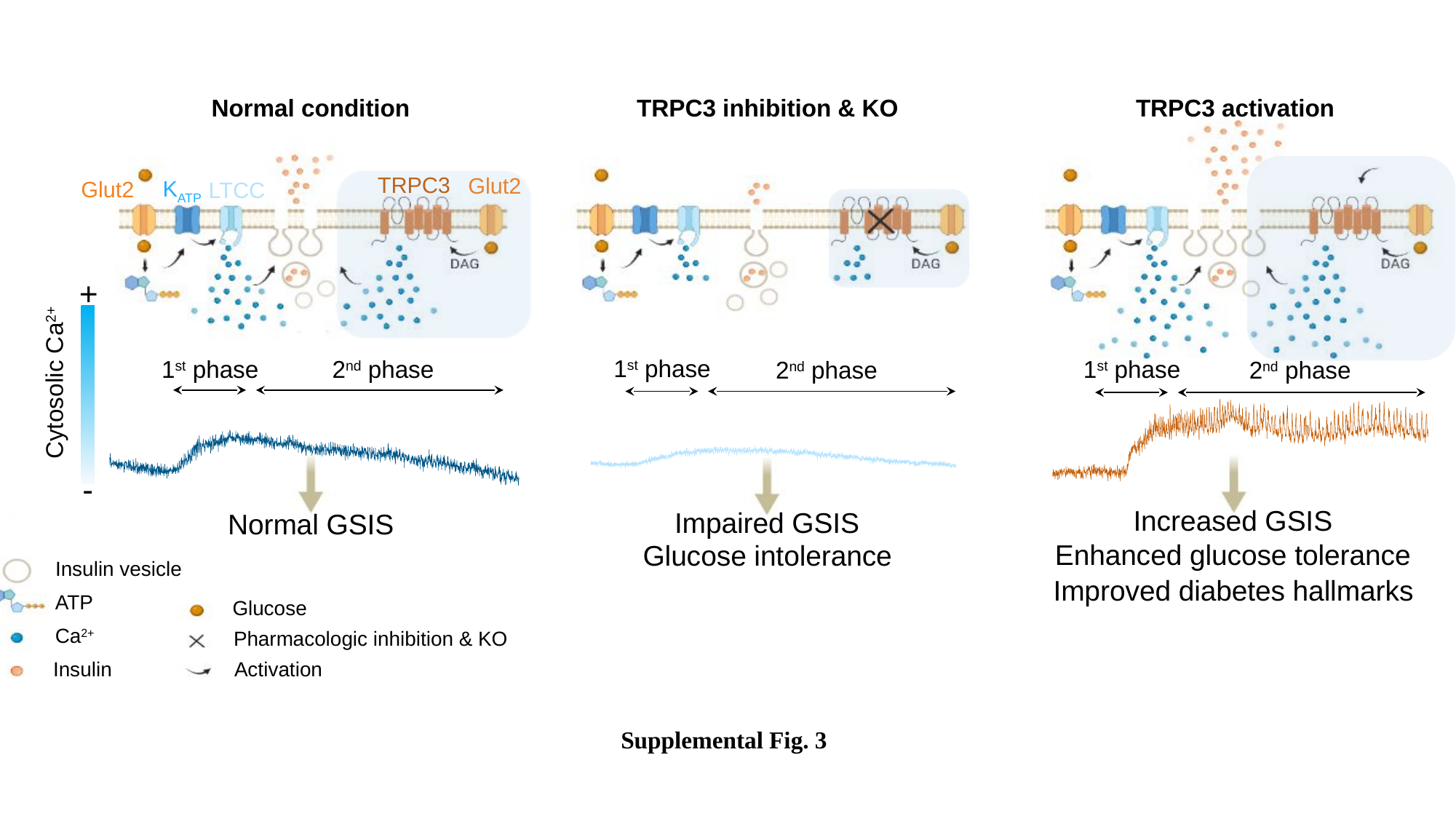

### Chart
| Category | Object[28] |
|---|---|Normal condition
TRPC3 inhibition & KO
TRPC3 activation
### Chart
| Category | Object[16] |
|---|---|TRPC3
Glut2
KATP
Glut2
LTCC
+
### Chart
| Category | Object[18] |
|---|---|1st phase
2nd phase
1st phase
2nd phase
1st phase
2nd phase
Cytosolic Ca2+
-
Increased GSIS
Impaired GSIS
Normal GSIS
Enhanced glucose tolerance
Glucose intolerance
Insulin vesicle
ATP
Ca2+
Insulin
Improved diabetes hallmarks
Glucose
Pharmacologic inhibition & KO
Activation
Supplemental Fig. 3
